# Distribution and species identification in the crustacean isopod genus *Dynamene* Leach, 1814 along the North East Atlantic-Black Sea axis

**DOI:** 10.3897/zookeys.635.10240

**Published:** 2016-11-23

**Authors:** Pedro E. Vieira, Henrique Queiroga, Filipe O. Costa, David M. Holdich

**Affiliations:** 1 Departamento de Biologia and CESAM - Centro de Estudos do Ambiente e do Mar, Universidade de Aveiro, Campus Santiago, 3810-193 Aveiro, Portugal; 2 CBMA - Centro de Biologia Molecular e Ambiental, Departamento de Biologia, Universidade do Minho, Campus Gualtar, 4710-057 Braga, Portugal; 3 Aquatic Consultant, Keyworth, Nottinghamshire, England, UK

**Keywords:** Dynamene, Crustacea, Isopoda, Sphaeromatidae, identification, distribution

## Abstract

Sphaeromatid isopods, such as *Dynamene*, are common and abundant members of the invertebrate fauna of littoral and shallow sublittoral substrates. Six species of *Dynamene* occur in the northern hemisphere. Only two species exist outside this range, in Australia. The distribution of the various species in the NE Atlantic-Black Sea axis has been controversial due to the difficulty in the identification of the different species. This has led to inaccurate records of their distribution, ultimately generating uncertain or faulty assessments on the biodiversity of these habitats. An update and a clarification about the distribution of this genus is therefore in order. In this study, we describe the distribution of *Dynamene* species in the light of new records from the NE Atlantic Ocean and its associated islands, and the Mediterranean, Black and Red Seas, and from re-examination of museum and several authors’ personal collections. Based on these observations, we extend the northern and southern limits of *Dynamene bidentata* (Adams); the western and southern limits of *Dynamene magnitorata* Holdich; the northern, eastern and western limits of *Dynamene edwardsi* (Lucas); and the eastern and western limits of *Dynamene bifida* Torelli. The range of *Dynamene tubicauda* Holdich is extended, but is still only known from the eastern Mediterranean. We also clarify the synonymy of *Dynamene torelliae* Holdich with *Dynamene bicolor* (Rathke), and the occurrence of *Dynamene bicolor* in the Black Sea. New distribution maps of the six *Dynamene* species are presented. Illustrated keys to the adult males and females of the northern hemisphere species are provided.

## Introduction

Isopod crustaceans are common and sometimes abundant members of the invertebrate fauna of the littoral and shallow sublittoral habitats of the world’s oceans (Poore and Bruce 2012). Species of the sphaeromatid isopod genus *Dynamene* Leach, 1814 are typical components of these habitats on coasts of the NE Atlantic Ocean and its islands, and the Mediterranean and Black Seas. Six species are endemic to these provinces (Holdich 1968a, 1970): *Dynamene bidentata* (Adams, 1800); *Dynamene bicolor* (Rathke, 1837); *Dynamene edwardsi* (Lucas, 1849); *Dynamene bifida* Torelli, 1930; *Dynamene magnitorata* Holdich, 1968 and *Dynamene tubicauda* Holdich, 1968. *Dynamene torelliae* Holdich, 1968 was considered to be synonymous with *Dynamene bicolor* by Kussakin (1979) and this has been accepted by the current authors. Two additional species occur in, and are endemic to, Australia, but have rarely been recorded: *Dynamene ramuscula* (Baker, 1908) and *Dynamene curalii* Holdich and Harrison, 1980. A number of other *Dynamene* species are incorrectly listed in some databases, e.g., http:/isopods.nhm.org/, Brusca et al. (1995-2004), Myers et al. (2008). Species attributed to the genus *Dynamene* from the western USA, i.e., *Dynamene angulata* Richardson, 1901; *Dynamene benedicti* (Richardson, 1899); *Dynamene dilatata* Richardson, 1899; *Dynamene glabra* Richardson, 1899 and *Dynamene sheari* Hatch, 1947 do not belong to this genus, as adult males do not possess a bidentate process arising from the sixth pereonite (see below), and are considered *incertae sedis* (http://www.marinespecies.org/). *Dynamene tuberculosa* Richardson, 1899 from the Aleutian Islands off Alaska is also still listed as such in some databases, but was considered as the female of *Paracerceis cordata* (Richardson, 1899) by Richardson (1905).

The distribution of the various *Dynamene* species associated with the NE Atlantic-Black Sea axis was previously examined by Holdich (1968a, 1970). Since then many general community studies have been published reporting the presence of *Dynamene* throughout its range (e.g., Pereira et al. 2006 in Portugal, Arrontes and Anadón 1990, Arrontes 1991, Viejo 1997, Castelló and Carballo 2001 in Spain, Castellanos et al. 2003 in northern Africa; and Kirkim et al. 2006 in Turkey). In addition, a large number of specimens have become available since Holdich’s studies, which make the clarification and updating of distribution maps along the NE Atlantic-Black Sea axis necessary. This is particularly so because many of the records for the Mediterranean and Adriatic relate to *Dynamene torelliae*, which has been synonymized with *Dynamene bicolor*.

**Figure 1.**
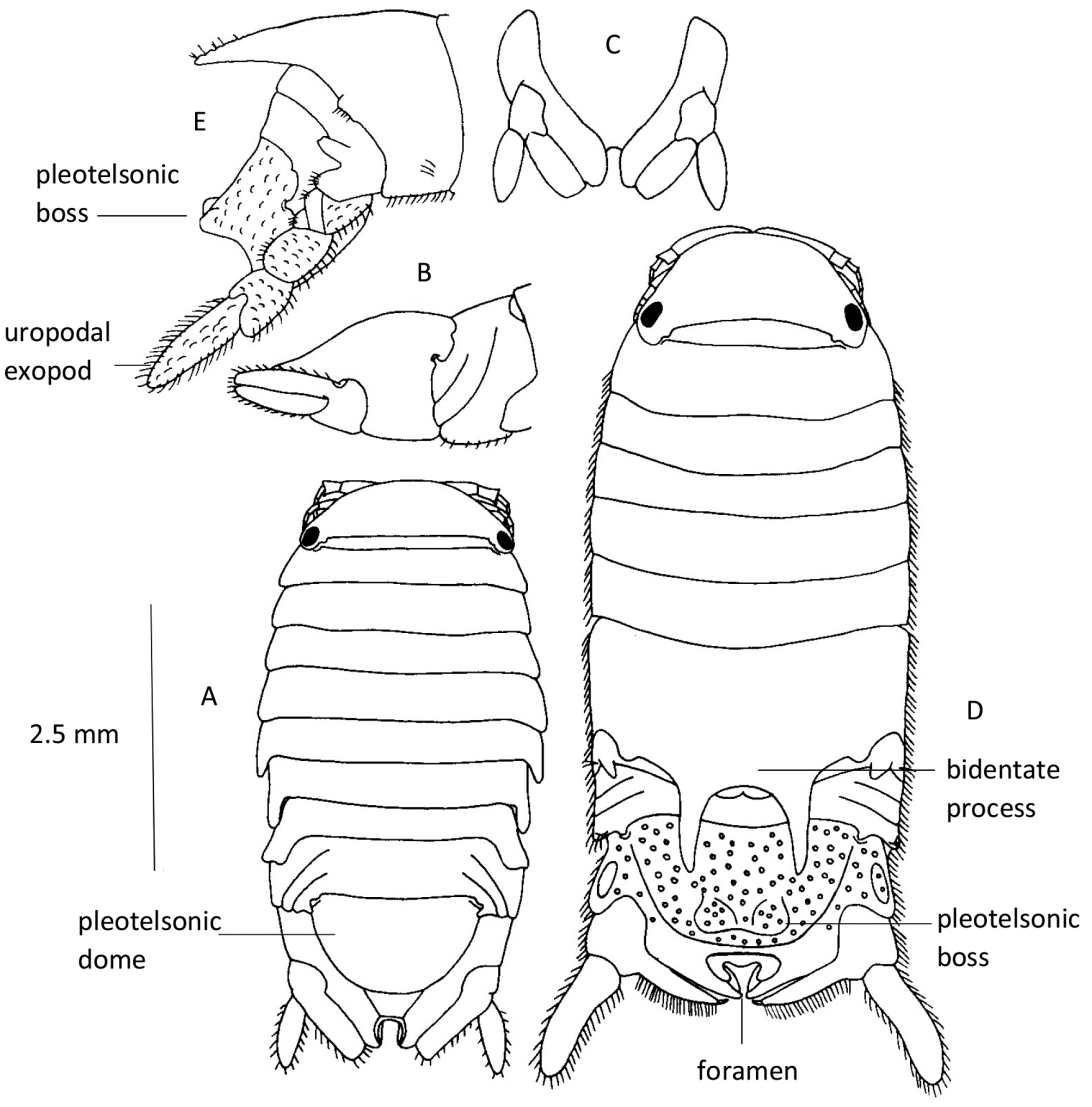
Adult male (stage 8) and pre-ovigerous female (stage 7) *Dynamene bidentata*. **A** Dorsal view of stage 7 female **B** Lateral view of pleon (with posterior border of pereonite 7), pleotelson and right uropod of stage 7 female **C** Ventral view of pleotelson and uropods of stage 7 female **D** Dorsal view of stage 8 male **E** Lateral view of pereonal segment 6, pleon, and pleotelson and exopod of right uropod of stage 8 male. Adapted from Holdich (1968b).

In order to be able to identify species of *Dynamene*, and distinguish them from some other sphaeromatid isopods, it is important to understand how the morphology changes during the life history. Adult males (stage 8) of the various *Dynamene* species can be distinguished from those of other sphaeromatid isopods, e.g., *Campecopea* Leach, 1814; *Cymodoce* Leach, 1814; *Ischyromene* Racovitza, 1908; *Lekanosphaera* Verhoeff, 1943 and *Sphaeroma* Bosc, 1802, that may be found in the same habitat, by a large two-pronged medial process (the bidentate process) arising from the dorsal posterior margin of the sixth pereonite (Fig. 1). This characteristic is unique to the genus (Harrison and Ellis 1991). Some species of *Oxinasphaera* Bruce, 1997 have such a process, but this arises from the pleon (Bruce 1997, Schotte and Kensley 2005), and paired processes arise from the seventh pereonite in *Dynamenella dioxus* Barnard, 1914. Juveniles and females, and even sub-adult males (stages 6 and 7), are more difficult to distinguish between the species, and may also be confused with females of other genera. Vieira et al. (2015) have shown clear differences between *Dynamene bidentata*, *Dynamene magnitorata* and *Dynamene edwardsi* at the genetic level using cytochrome oxidase I (COI-5P). Details of the changes occurring throughout the life history of the best-studied species, *Dynamene bidentata*, are given below.

*Dynamene* species are present in a wide-range of habitats, but usually amongst algae and in cryptic habitats, e.g., under rocks, crevices, empty barnacle tests, amongst serpulid and tunicate colonies, mussel beds and encrusting sponges, from midlittoral to shallow sublittoral levels (Holdich 1970, 1976). *Dynamene bidentata*, at least, has a biphasic life cycle with a change of habitat, where the immature stages are present amongst the algal cover (which they eat), whilst the adults occupy cryptic habitats where they reproduce and where females can incubate their broods in relative safety (Holdich 1968b, 1970, 1976). Further details of the habitats occupied by *Dynamene* along the NE Atlantic-Black Sea axis are given for each species below.

Given that fully adult males may not be present in many collections, species identification is often difficult and leads to incorrect assignments, questioning the validity of the information about the actual distribution of the species. The literature is scattered with misidentifications, which have come to light when such authors’ material and/or publications have been examined by us. In the present study we aim to update and correct the geographical distribution of the six described species of *Dynamene* from the NE Atlantic-Black Sea axis. To facilitate identification, keys to adult males and females of these six species are provided along with associated photographs. It is hoped that these will enable those involved in littoral and sublittoral surveys in the marine environment to identify species of *Dynamene* more easily.

## Methods

The records of David Holdich (DMH) used in this study are derived from field work carried out in various localities in the British Isles, Atlantic islands, Atlantic coasts of mainland Europe, and the Mediterranean and Aegean Seas (Holdich 1968a, c, 1970, Holdich and Lincoln 1974, Holdich 1976). In addition, there have been donations from many colleagues between 1970 and 2014 (see Acknowledgments). Other samples deposited in several museum collections, particularly those in Leiden, Lisbon, London and Paris (see Acknowledgements), and dating back to the 1920s, have been examined. Also, the *Dynamene* specimens (deposited at the Universities of Aveiro and Minho) collected by Pedro Vieira, Henrique Queiroga and Filipe Costa with the help of other colleagues (see Acknowledgments) were used to supplement the collections. These samples were collected from the NE Atlantic coasts and the Macaronesian archipelagos of Madeira, Azores and Canary islands, between 2009 and 2015. Samples were taken from rocky shore habitats by scraping of the algal cover and hand picking during low tide.

All specimens of *Dynamene* from DMH’s collections have been deposited in the Naturalis Biodiversity Centre, Leiden, The Netherlands under the catalogue numbers: RMNH.CRUS.1. 7517-7616 and 7642-7676. Specimens of *Dynamene* already present in the Leiden collections have the catalogue numbers: RMNH.CRUS.1. 7450-7514.

In most cases the only records considered were of specimens actually seen by the authors, confirmed by molecular tools (unpublished data), or where there were clear diagrams in the literature. Although Holdich (1968c) confirmed many specimens from England and Wales during his surveys, since that time most records of *Dynamene bidentata* have mainly come about as part of the general fauna collected in marine surveys. So, although many records exist in various British databases, particularly those held in the National Biodiversity Network Gateway and ERICA (see Acknowledgements), the current authors have not tried to track down voucher specimens, but have relied on identifications being correct as only one species of *Dynamene* is indigenous to the British Isles, thus making records more reliable. Details of all the specimens examined in the current study are given in Suppl. material 1.

Using information in the databases, maps were constructed of the six *Dynamene* species occurring along the NE Atlantic-Black Sea axis using the software ARCGIS 10.3.

Keys and photographic montages based on the main characters of adult males (stage 8) and females are given at the end of the paper. To construct the montages, photographs of alcohol preserved specimens were taken with a Dino-Eye Microscope Camera attached to a Wild M5 binocular microscope via a phototube. Images were edited using appropriate software on a computer.

## Results

In this section a generic description of *Dynamene* is given, followed by details of each of the six species present along the North East Atlantic-Black Sea axis. Keys to and photographs of males and females of each species are given at the end of the paper. Comparisons are made in the main discussion section and overall conclusions are dealt with in the final section. Details of the material examined and geographical coordinates of locations are given in Suppl. materials 1 and 2.

### Dynamene

Taxon classificationAnimaliaIsopodaSphaeromatidae

Leach, 1814

#### Synonymy.

*Nesaea*: Leach (1814).

*Prochonaesea*: Hesse (1873).

*Sorrentosphaera*: Verhoeff (1944).

#### Diagnosis.

Eubranchiate sphaeromatid with body approximately elliptical. Anteriorly, cephalosome separating the bases of the antennules. Eyes set slightly into pereonal tergite 1. Coxal plates of pereonites 1–7 separated from tergites by sutures.The seventh somite is overlapped by the sixth in adult males (stage 8), with the pleura extended postero-laterally into two small processes, which vary in shape according to species. Pleotelson domed or keeled, and terminating in an obvious terminal foramen, which may be enclosed forming a tube. Antennular peduncle articles 1 and 2 dilated and juxtaposed to ventral margins of cephalosome. All pereopods ambulatory. Both rami of pleopods 1-3 bearing margin of plumose setae. Endopods of uropod fused with protopods and juxtaposed to pleotelsonic margin; exopods posteriorly directed. Sexual dimorphism pronounced. Adult male with pereonal tergite 6 longer than those preceding, posterior margin with an elongate, posteriorly directed process either side of the mid-line (the bidentate process). Posterior part of pleotelson with central boss. Penes small, separate. Endopod of pleopod 2 lacking appendix masculina. Female with pereonal tergite 7 similar to those preceding and lacking bidentate process; pleotelson smooth. Ovigerous female with ventral marsupium, formed from four pairs of lamellae, which arise from pereonites 1-4. Mouthparts strongly metamorphosed.

#### Type species.

*Oniscus bidentatus* Adams, 1800

### Dynamene bidentata

Taxon classificationAnimaliaIsopodaSphaeromatidae

(Adams, 1800)

#### Restricted synonymy.

*Oniscus bidentatus*: Adams (1800).

*Naesa bidentata*: Leach (1815).

*Dynamene bidentata*: Holdich (1968a, b, c, 1969, 1970, 1971, 1976); Kussakin (1979); Harrison and Ellis (1991).

An extensive synonymy was given by Holdich (1968a, c) for citations prior to 1968.

#### Material examined.

Specimens have been examined from 129 locations in the NE Atlantic, mainly from the British Isles, Channel Islands, France, Spain, Portugal and Morocco – see the Suppl. materials 1 and 2. A number of literature records have been included where the diagrams clearly indicate this species. In addition, there are 76 records from the NBN database.

#### Key morphological characters.

**Figure 2.**
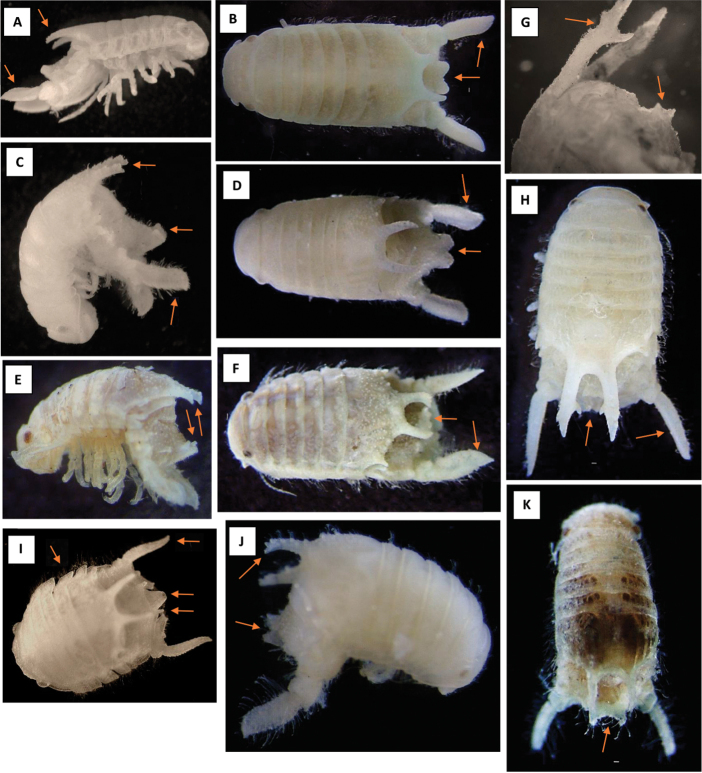
Main features of adult males (stage 8) of the NE Atlantic-Black Sea axis *Dynamene* spp. **A, B** *Dynamene bidentata* (S. Wales). Arrows indicate shape of the bidentate process (**A**), uropods (**A, B**) and pleotelsonic boss (**B**) **C, D** *Dynamene magnitorata* (Roscoff, France). Arrows indicate shape of the bidentate process (**C**), the uropods (**C, D**) and the pleotelsonic boss (**C**, **D**). Note the difference in the shape of the boss and the ends of the arms of the bidentate process to those of *Dynamene bidentata* **E, F** *Dynamene edwardsi* (**E** Canaries **F** Azores). Arrows indicate shape of the bidentate process (**E, F**), uropods (**F**) and pleotelsonic boss (**E, F**). Specimen in E shows relatively little dorso-lateral setation, whilst that in **F** is hirsute. Note the differences in the shape of the boss and the tips of the arms of the bidentate process compared to those of *Dynamene bidentata* and *Dynamene magnitorata* **G, H** *Dynamene bifida* (France, Mediterranean). Arrows indicate shape of the bidentate process (**G, H**), uropodal exopod (H) and pleotelsonic boss (**G**). Note the large accessory process on each arm of the bidentate process, the small sessile pleotelsonic boss and the long narrow uropodal exopods **I** *Dynamene tubicauda* (Bay of Naples, Italy). Arrows indicate the unique body shape, tubular respiratory channel, peg-like pleotelsonic bosses, and the curved uropodal exopods **J, K** *Dynamene bicolor* (Bay of Naples, Italy). Arrows indicate shape of the bidentate process (**J**), and pleotelsonic boss (**J, K**). Note in particular the rugose nature of the dorsal surface of the bidentate arms, and the triangular shape of each half of the boss – in specimens from the Black Sea the boss is of a similar shape but much less prominent.

**Figure 3.**
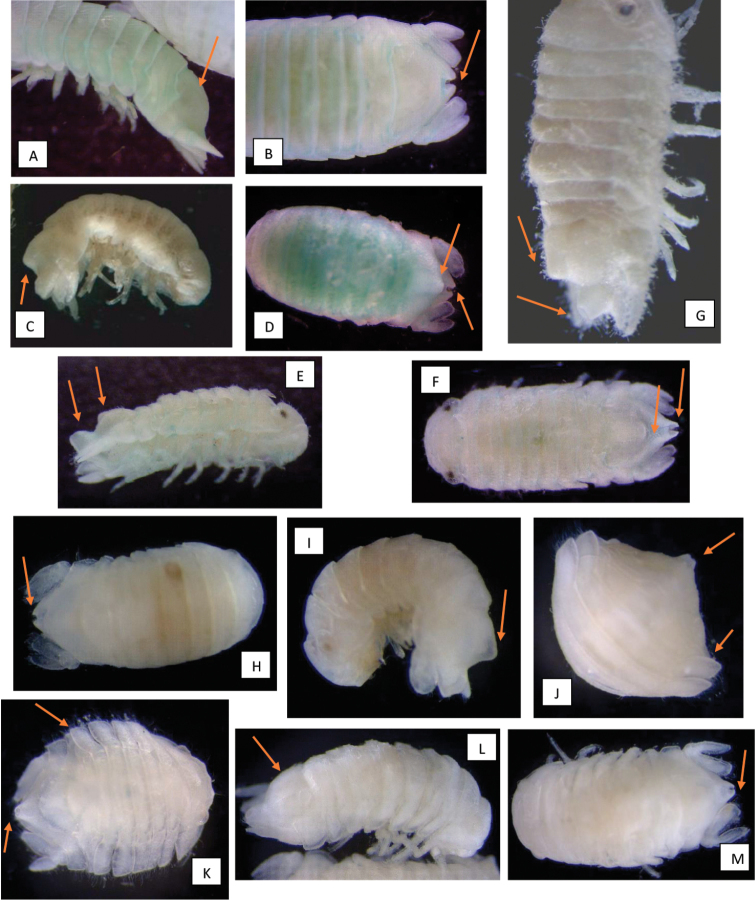
Main features of females and juveniles of the NE Atlantic-Black Sea axis *Dynamene* spp. **A, B** *Dynamene bidentata* (S. Wales). Arrows indicate smooth outline of pleotelsonic dome (**A**) and non-tubular pleotelsonic foramen (**B**) **C, D** *Dynamene magnitorata* (Roscoff, France). Arrows indicate angular outline of pleotelsonic dome (**C**), posterior extension of pleotelsonic keel and non-tubular pleotelsonic foramen (**D**) **E, F, G** *Dynamene edwardsi* (Italy). Arrows indicate angular outline of pleotelsonic dome (**E**) with central bulge (**E, F, G**) and tubular pleotelsonic foramen. (**E** and **F** from Naples, Italy **G** hirsute female from the Venice Lagoon, Italy) **H, I** *Dynamene bicolor* (Naples, Italy). Arrows indicate angular outline of pleotelsonic dome (**I**) and non-tubular pleotelsonic foramen (**H**) **J**, **K** *Dynamene tubicauda* (Ischia, Italy). Arrows indicate flattened epimera surrounding body that give this species a unique body shape (**J, K**) and the tubular pleotelsonic foramen (**J, K**) **L, M**. *Dynamene bifida* (Ischia, Italy). Arrows indicate smooth outline to pleotelsonic dome (**L**) and pleotelsonic foramen at end of short tube (**M**).

Body convex; in stage 8 males the pleotelsonic boss is large and bilobed, the two halves are separated by a wide v-shaped groove; the arms of the bidentate process taper to a point, and are sparsely rugose dorsally (Fig. 2A–B). In stage 7 females the pleotelsonic dome is smoothly rounded in side view and the pleotelsonic foramen is open and flush with the edge of the pleotelson (Fig. 3A–B). In populations from Atlantic coasts the smooth outline of the pleotelsonic dome in females and juveniles is key to separating this species from *Dynamene magnitorata* and *Dynamene edwardsi*, where it is keeled in side view. Further details are provided by the scanning electron microscope pictures of the posterior body of a stage 8 male and a stage 7 female in Holdich (1976).

#### Size.

Adult males (stage 8) typically 7.0 × 3.0 mm, although specimens 10 mm in length have been seen; pre-ovigerous females (stage 7) typically 6.0 × 2.9 mm.

#### Life-history.

**Figure 4.**
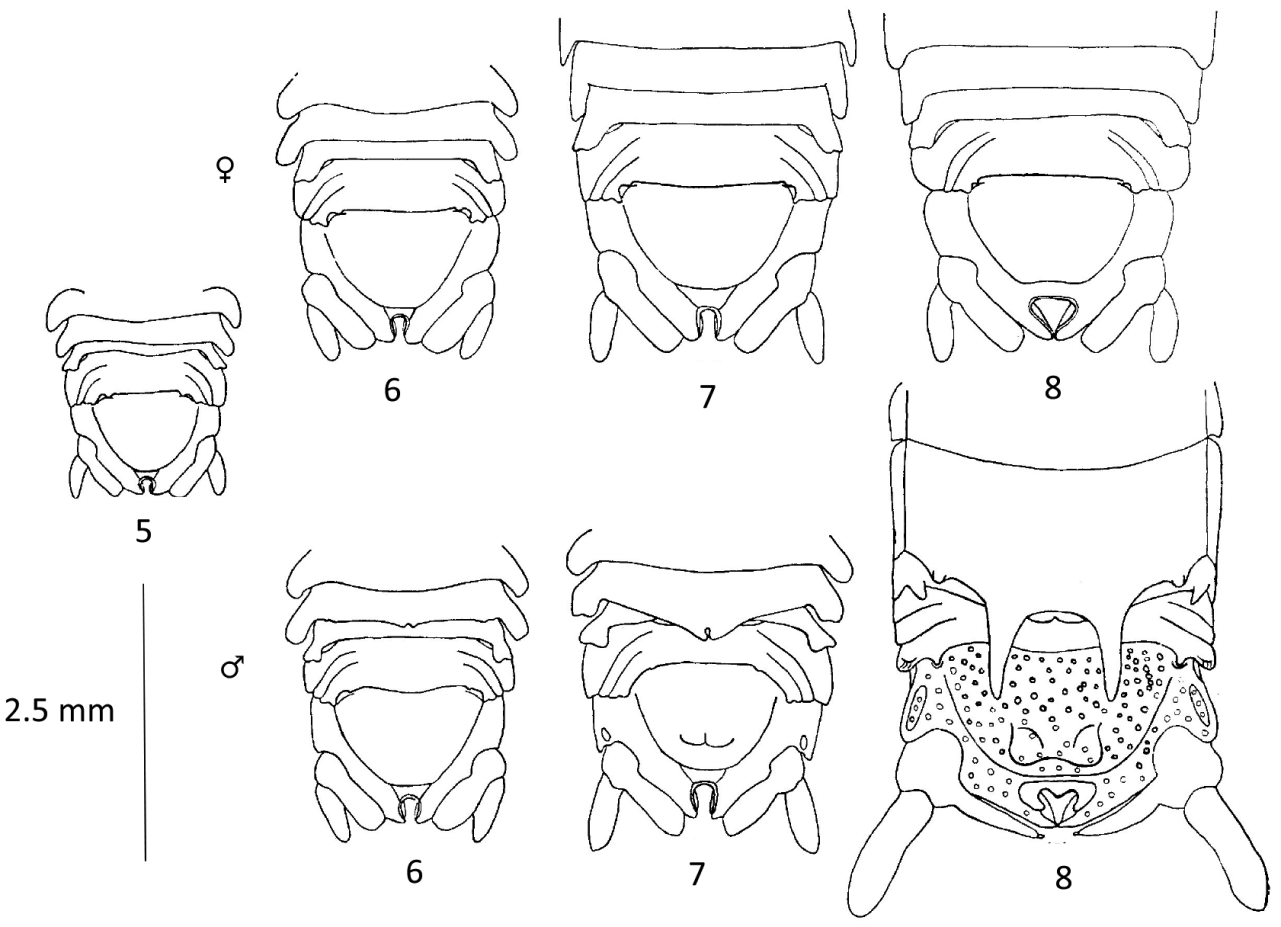
Dorsal views of the posterior halves of the bodies of various life history stages (**5–8**) of *Dynamene bidentata*. **5** juvenile **Upper row** female stages **6, 7, 8** (ovigerous) **Lower row** male stages **6, 7, 8**. Adapted from Holdich 1968b.

There are eight life-history stages in both males and females (Holdich 1968b). Sexual dimorphism becomes apparent in stage 6 males with the appearance of a very small bidentate process, this increases in size at the seventh, and is fully developed by the eighth and terminal stage (Figs 1D, 4–lower row 6–8). This process is absent from juveniles and females (Figs 1A-B, 4–upper row 6–8, 2A–M). Juveniles and females up to and including stage 7 are very similar to each other morphologically. At the moult to stage 8 females become ovigerous and are very similar morphologically between the species. Their mouthparts are strongly metamorphosed, and they die after releasing their broods (Hansen 1905, Holdich 1968b, 1971). Stage 8 males live for two breeding seasons, at least in the British Isles, and remain in their cryptic habitat for the entire period without apparently feeding (Holdich 1971). Those in their second year are recognizable from the growths of algae, and sometimes serpulids, on the pleotelson.

#### Habitat.

All stages can be found on a wide variety of mid- to lower littoral algae, and also in rock pools in the upper littoral zone. Fenwick (pers. comm., July 2016) has found this species commonly amongst lower shore and sublittoral coralline algae in Cornwall, and he has also recorded adults from under large lower shore pebbles. Stage 7 females and stage 8 males move from the algae into cryptic habitats, such as crevices and empty barnacle tests, particularly *Balanus perforatus*, to breed (Holdich 1970, 1976). Stage 7 females moult into stage 8 females within such a habitat and reach peak numbers in April/May each year (Holdich 1968b).

#### Colour.

Some degree of camouflage in the algal habitat is given by green, yellow and brown ‘uniformis’ phenotypic varieties, and this is enhanced by the development in some individuals of patterns of white or red, dorsal, non-adaptable chromatophores (Tinturier-Hamelin 1962, 1967, Holdich 1969, Arrontes 2009). In the past some workers have given specific status to the red and green colour varieties, e.g. rubra and viridis (see Holdich 1968c). Adult males are particularly colourful when found amongst red algae on the lower shore, with the margins of the body segments and uropods bordered in orange.

#### Geographical distribution.

**Figure 5.**
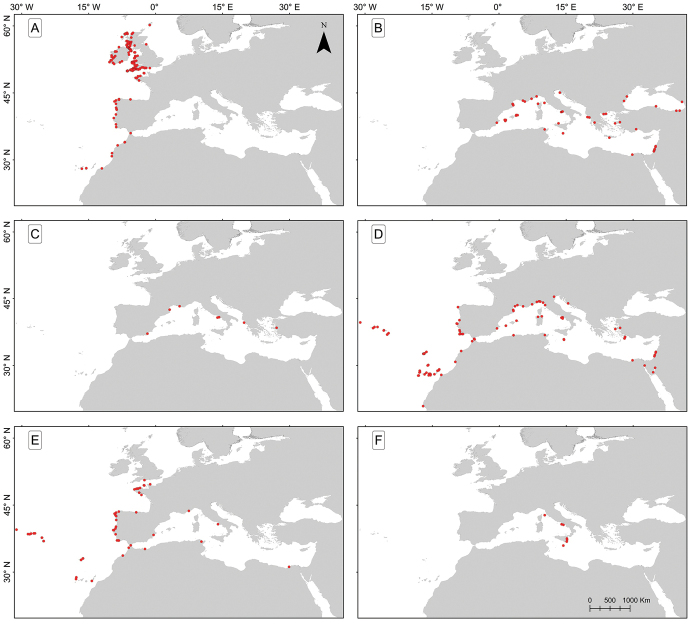
Distribution of *Dynamene* species along the NE Atlantic-Black Sea axis based on material validated during the present study. **A** *Dynamene bidentata* **B** *Dynamene bicolor* **C** *Dynamene bifida* **D** *Dynamene edwardsi* **E** *Dynamene magnitorata* **F** *Dynamene tubicauda*.

The distribution of this species shown in Holdich (1970, 1974) has been extended by the present study. It occurs from the Shetland Islands to Tarfaya in western Morocco and Tenerife and Gran Canaria in the Canary Islands, which are the only two records of the species in Macaronesia (Fig. 5A). Within this range *Dynamene bidentata* occurs in the north, northwest (including the outer islands), west and south coasts (as far as the Isle of Wight) of Great Britain, around Northern and Southern Ireland, the Channel Islands, northwest France, Atlantic Iberian Peninsula and in northwest Africa. Arrontes (1991) cites *Dynamene bidentata* as being the most abundant isopod species on shores in northern Spain. It is the only species present in the British Isles (with the exception of a single record of *Dynamene magnitorata* in southern England). It is particularly common in SW England and SW Wales, especially where the large barnacle, *Balanus perforatus* is present. There is one recent record for north-eastern England, which may be the result of a stranding, as are records for The Netherlands, where it is not considered indigenous (Holthuis 1956). The closest record to the Mediterranean of *Dynamene bidentata* is Tarifa, in southern Spain (Guerra-García et al. 2011, Izquierdo and Guerra-García 2011, Guerra-García et al. 2012, Torrecilla-Roca and Guerra-García 2012).

#### Remarks.

Maggiore and Fresi (1984) described *Dynamene bidentata* from the Gulf of Naples (publishing descriptions and figures), and several authors (e.g., Castelló and Carballo 2001, Castellanos et al. 2003, Junoy and Castelló 2003) have used Maggiore and Fresi’s (1984) observations to justify their findings of *Dynamene bidentata* in the Mediterranean. Yet, examination of the single specimen found by Maggiore and Fresi (1984) showed that it was in fact a *Dynamene magnitorata*.

A lot of confusion regarding the identification of *Dynamene bidentata* was caused by Torelli (1930) who figured what she called *Dynamene bidentata* (a stage 8 male and a stage 8 ovigerous female), from the Bay of Naples, Italy. Omer-Cooper and Rawson (1934) used Torelli’s figures to illustrate *Dynamene bidentata* from Britain, which was then proliferated in some British identification guides, e.g., Barrett and Yonge (1964), although this has been corrected in more modern guides, e.g., Hayward and Ryland (1995). Pauli (1954) also used Torelli’s figures to illustrate *Dynamene bidentata* from the Black Sea. Holdich (1968a) collected material from Naples and decided that Torelli’s figures were in fact of a new species, commonly found in the Bay of Naples, which he named *Dynamene torelliae* Holdich, 1968. However, Kussakin (1979) decided that *Dynamene torelliae* was in fact synonymous with *Dynamene bicolor* (Rathke, 1837). This species was in fact unknown to Holdich at the time of his studies.

Databases we have consulted indicate that *Dynamene bidentata* commonly occurs around Northern and Southern Ireland. However, we could only find one modern published record, i.e., de Grave and Holmes (1998) from Lough Hyne in County Cork.

Unlike most other isopods, stage 8 male *Dynamene bidentata* do not have appendix masculina on the endopods of the second pair of pleopods, this is also the case in the other *Dynamene* species. This phenomenon has also been noted by Messana (2004) in *Sphaeroma terebrans* Bate, 1866. It is very difficult to observe mating in *Dynamene* due to the cryptic habitat of the adults. It is probable that sperm are released directly into the marsupium as the eggs are laid.

### Dynamene bicolor

Taxon classificationAnimaliaIsopodaSphaeromatidae

(Rathke, 1837)

#### Restricted synonymy.

*Campecopea bicolor*: Rathke (1837).

*Dynamene bidentata*: Torelli (1930); Omer-Cooper and Rawson (1934); Pauli (1954); Holthuis (1956); Barrett and Yonge (1964); [not *Dynamene bidentata* of Adams (1800)].

*Dynamene torelliae*: Holdich (1968, 1970).

*Dynamene bicolor*: Kussakin (1979); Maggiore and Fresi (1984).

#### Material examined.

Specimens have been examined from 48 locations in 12 countries in the Mediterranean and Black Seas - see the Suppl. materials 1 and 2. A number of literature records have been included where the diagrams clearly indicate this species.

#### Key morphological characters.

In stage 8 males the pleotelsonic boss is comprised of two right-angled triangular structures separated by a deep groove (however, the boss may be very low lying in some specimens, e.g., those from the Black Sea); the arms of bidentate process taper to a point and are rugose dorsally (Fig. 2J–K). In stage 7 females the pleotelsonic dome is keeled in side view and the pleotelsonic foramen is flush with the edge of the pleotelson (Fig. 3H, I). The females of this species are very difficult to separate from those of *Dynamene magnitorata*. Maggiore and Fresi (1984) provide a complete description of *Dynamene bicolor*.

#### Size.

Adult males (stage 8) typically 3.5 × 1.5 mm, pre-ovigerous females (stage 7) typically 3.0 × 1.3 mm.

#### Life-history.

Nothing is known of the life-history, other than the fact that sexual dimorphism occurs with males developing the bidentate process characteristic of the genus.

#### Habitat.

Juveniles are usually found in shallow water on a variety of algae down to 3.0 m and adults in empty *Balanus* tests, in mussel beds, in rock crevices, within sponges, and under rocks throughout the Mediterranean. However, occasionally they have been found in deeper water, e.g., off the island of Chios (Greece) specimens were collected from *Cystoseira* at depths from 0.5 – 30 m (see Suppl. material 1).

#### Colour.

As with *Dynamene bidentata*, some degree of camouflage in the algal habitat is given by yellow or dull green ‘uniformis’ phenotypic varieties, and this is enhanced by the development in some individuals of patterns of white or red, dorsal, non-adaptable chromatophores (Holdich 1969).

#### Geographical distribution.

The distribution of this species shown in Holdich (1970) has been extended by the present study. It is the most commonly recorded *Dynamene* species in the Mediterranean, occurring from the Balearic Islands in the west to the coast of Israel in the east, although there are only a few records for the North African coast (Fig. 5B). It has been frequently recorded around the Greek islands and mainland coast of both Greece and Turkey. The most northerly record is for Croatia in the Aegean Sea. It has also been recorded for a number of countries around the Black Sea (Bulgaria, Romania, Turkey and Georgia) (Fig. 5B). Most records in the literature refer to *Dynamene torelliae*, which is now considered synonymous with *Dynamene bicolor*.

#### Remarks.

Many records exist, both published and unpublished, for *Dynamene bicolor* (usually as ‘*Dynamene torelliae*’) in the Mediterranean Sea, particularly from the coasts of Spain, France, Italy and Greece (Holdich 1970, Bakir et al. 2014). However, its presence in Egypt and Israel was unreported until now. Previous observations indicated its presence in the Black Sea (Kussakin 1979), where it was thought to be the only *Dynamene* species present (Gönlügür-Demirci and Katağan 2004). On comparing specimens from the Black and Mediterranean Seas the current authors have accepted the decision of Kussakin (1979) that *Dynamene torelliae* and *Dynamene bicolor* are synonymous. However, it is clear that some of the specimens from the Black Sea have a reduced pleotelsonic boss, and the two may eventually turn out to be separate species when more material is examined. Kirkim et al. (2006) commented on the form of the pleotelsonic boss, stating that this can vary from two small projections to a well-formed boss in specimens of ‘*Dynamene torelliae*’ from the Aegean Sea. Rathke’s (1837) drawings of *Dynamene bicolor* show the posterior halves of a female and a stage 7 male. The male has two joined hemispherical pleotelsonic bosses, which are similar to those found in the same stage of ‘*Dynamene torelliae*’ and unlike that of *Dynamene edwardsi* the other species in the region, which is single.

### Dynamene bifida

Taxon classificationAnimaliaIsopodaSphaeromatidae

Torelli, 1930

#### Restricted synonymy.

*Dynamene bifida*: Torelli (1930).

*Dynamene bifida*: Holdich (1968, 1970).

#### Material examined.

Specimens were examined from seven locations in Spain, Greece, France, Italy and Turkey in the Mediterranean – see the Suppl. materials 1 and 2. A number of literature records have been included where the diagrams clearly indicate this species.

#### Key morphological characters.

In stage 8 males each arm of the bidentate process is large, tapering and with a well-developed, downwardly-directed accessory process a quarter of the way from the apex; the pleotelsonic boss is very small with raised pointed corners (Fig. 2G–H). In stage 7 females the pleotelsonic dome is smoothly rounded in side view and the pleotelsonic foramen is at the end of short tube (Fig. 3L–M).

#### Size.

Adult males (stage 8) typically 5.0 × 3.0 mm, although a specimen of 7.0 mm length has been seen; pre-ovigerous females (stage 7) typically 4.0 × 2.0 mm.

#### Life-history.

Nothing is known of the life-history of this species, other than the fact that sexual dimorphism occurs with males developing the bidentate process characteristic of the genus.

#### Habitat.

Adults, including stage 8 females, were found among *Hydroides unicata* colonies and other cryptic habitats in the Bay of Naples (Torelli 1930, Holdich 1970). Ledoyer (1962) recorded it from *Ulva lactuca* at Endoume, southern France, and Holthuis (unpublished records) from rocky shores amongst algae at 0.0–1.0 m at Banyuls-sur-Mer. The latter record included stage 8 females.

#### Colour.

All specimens seen were a pale, sandy yellow. No polychromatism was observed.

#### Geographical distribution.

The distribution of this species shown in Holdich (1970) has been extended by the present study. It has a widespread distribution in the Mediterranean stretching from southern Spain to Turkey (Fig. 5C).

#### Remarks.

Originally described by Torelli (1930) from the Bay of Naples, males of this distinctive, and sometimes large, species has been infrequently recorded, and females even less so. The accessory process on each arm of the bidentate process is similar to that found in the Australian species, *Dynamene ramuscula* (Holdich and Harrison 1980). The fact that ovigerous females were found amongst shallow-water algae raises questions about the life-history of this species, although in the Bay of Naples this stage has been recorded with males in more protective habitats.

### Dynamene edwardsi

Taxon classificationAnimaliaIsopodaSphaeromatidae

(Lucas, 1849)

#### Restricted synonymy.

*Naesa edwardsi*: Lucas (1849).

*Dynamene hanseni*: Monod (1923).

*Dynamene edwardsi*: Holdich (1968a, 1970); Harrison (1982).

*Dynamene bidentata*: Picker and Griffiths (2011).

An extensive synonymy was given by Holdich (1968a, c) for citations prior to 1968.

#### Material examined.

Specimens were examined from 89 locations in NE Atlantic, Mediterranean, Adriatic, Aegean and Red Seas – see Suppl. materials 1 and 2. A number of literature records, e.g., the Suez Canal, have been included where the diagrams clearly indicate this species.

#### Life-history.

Nothing is known of the life-history of this species, other than the fact that sexual dimorphism occurs with males developing the bidentate process characteristic of the genus.

#### Key morphological characters.

Body convex; in stage 8 males the apices of arms of the bidentate process are swollen, each with a short, downwardly-directed spur; the pleotelsonic boss is plate-like with two forward-facing pegs; the body exhibits various degree of setation (Fig. 2E–F), e.g., specimens examined from the Balearic Islands (Spain) and the island of Chios (Greece) are somewhat different from other *Dynamene edwardsi* seen by us in being very hirsute, with a pronounced developing boss and respiratory tube in the stage 7 males. In stage 7 females the pleotelsonic dome is keeled in side view, with a median protuberance; the pleotelsonic foramen is at the end of a short tube (Fig. 3E–G). Further details are provided by the scanning electron microscope pictures of the posterior body of a stage 8 male and a stage 7 female in Holdich (1976). See keys to stage 8 males and stage 7 females.

#### Size.

Adult males (Stage 8) typically 5.5 × 2.25 mm; pre-ovigerous females (stage 7) typically 3.0 × 1.1 mm, specimens of 4.4 × 2.3 mm have been seen from the Venice Lagoon, Italy.

#### Habitat.

Juveniles and adults have been found amongst a variety brown, green and red algae in the littoral and sublittoral zones, sometimes in conjunction with *Dynamene bicolor* in the Mediterranean, and with *Dynamene bidentata* and *Dynamene magnitorata* on Atlantic coasts. Adults have also been recorded from amongst mussels and tube worm colonies and barnacle tests in the Bay of Naples (Torelli 1930, Holdich 1970), and elsewhere in the Mediterranean (e.g., Rivosecchi 1961, Bellan-Santini 1962). It has been found associated with encrusting matter on solid surfaces in some harbours and canals. On occasions it has been found amongst the ‘trottoir’ on steep-sided cliffs as deep as 10 m. Monod (1932) recorded it from coralline and fucoid algae on the coast of NW Africa. In the Azores, adults have been recorded from empty *Chthamalus stellatus* tests attached to lower shore cobbles, along with *Campecopea lusitanica*. In West Portugal (Buarcos) it is present with *Dynamene bidentata* and *Dynamene magnitorata*. However, while *Dynamene bidentata* adults were present in barnacles, no *Dynamene edwardsi* were found inside barnacles, only among intertidal algae and on a few ‘small’ algae in shaded crevices at 0-1 m. Also, they were not present among mussels. Unusually, adults, including stage 8 females, were found in upper shore sandstone crevices, along with *Campecopea hirsuta*, in southern Portugal.

#### Colour.

The general body colour is a dull grey-green, individuals sometimes exhibit polychromatism caused by patterns of white, dorsal, non-adaptable chromatophores as seen in some of the other species (Holdich 1969).

#### Geographical distribution.

The distribution of this species shown in Holdich (1970) has been extended by the present study. It is the most meridional of the Atlantic species, occurring from Galicia in north-western Spain to Nouadhibou in Mauritania (Fig. 5D). This is the currently known southern limit of *Dynamene* species of the NE Atlantic-Black Sea axis. It is widespread in the Macaronesian islands and in the eastern and western Mediterranean (Fig. 5D). The most northerly record comes from the Venice Lagoon in the Adriatic Sea. It is also the only *Dynamene* species recorded from the Red Sea, in the Gulf of Aqaba (Fig. 5D). Glynn (1972) recorded a species that is clearly *Dynamene edwardsi* from the Suez Canal. Picker and Griffiths (2011) have recorded this species (as *Dynamene bidentata*) from South Africa.

#### Remarks.

*Dynamene edwardsi* occupies a wide vertical range in the littoral zone on NE Atlantic shores, and from the littoral zone down to 10 m in the Mediterranean. In recent field work, it was found to be very abundant in the Canary Islands and Madeira archipelago, whereas *Dynamene magnitorata* was more common in the Azores and *Dynamene edwardsi* rare. It is the most southerly of the *Dynamene* species extending down the West African coast to Mauritania and the only record for tropical waters. Glynn (1972) suggested that *Dynamene edwardsi* has migrated from the Mediterranean throughout the whole length of the canal. Our study has shown that it has now reached the Gulf of Aqaba in the Red Sea

The records for the Suez Canal and Red Sea are interesting as they show movement from the Mediterranean Sea into the Red Sea, whilst many marine species are moving in the opposite direction (Galil et al. 2014). No *Dynamene* species have yet been recorded from the Indian Ocean (Schotte and Kensley 2005). However, a stage 8 male has been recorded from Port Elizabeth harbour in South Africa by Picker and Griffiths (2011). They suggest that it may have been introduced as a fouling organism or in ballast water. It is known that this species can be transported amongst fouling organisms on ships, as evidenced by the finding a stage 8 male on a ship in Tangiers harbour (Morocco) (see Suppl. material 1).

This species is variable in its morphology and particularly in the degree of hirsuteness. It may be that some of the specimens collected from the Balearic and Greek islands are in fact a new species, but more material is needed to prove this. Ideally, a molecular genetic analysis needs to be carried out on Mediterranean and Adriatic specimens. Such a technique applied to specimens from some NE Atlantic coasts and Macaronesian islands has shown that a number of cryptic species may be present (Vieira et al. 2015).

### Dynamene magnitorata

Taxon classificationAnimaliaIsopodaSphaeromatidae

Holdich, 1968

#### Restricted synonymy.

*Dynamene magnitorata*: Holdich (1968).

*Dynamene bidentata*: Monod (1932); Maggiore and Fresi (1984).

*Dynamene magnitorata*: Holdich (1968a, 1970, 1976).

#### Material examined.

Specimens were examined from 52 locations in the NE Atlantic, and four countries in the Mediterranean – see the Suppl. materials 1 and 2. A number of literature records have been included where the diagrams clearly indicate this species.

#### Key morphological characters.

Body convex; in stage 8 males the pleotelsonic boss is large, bilobed, with the two halves separated by a narrow groove; the arms of the bidentate process are of similar width along their lengths and are dorsally tuberculate (Holdich 1976, fig. 3A, B; Fig. 2C–D in this paper). In stage 7 females the pleotelsonic dome is keeled in side view and the pleotelsonic foramen is flush with the edge of the pleotelson (Fig. 3C–D). Further details are provided by the scanning electron microscope pictures of the posterior body of a stage 8 male and a stage 7 female in Holdich (1976). The females of this species are very difficult to separate from those of *Dynamene bicolor*. See keys to stage 8 males and stage 7 females.

#### Size.

Adult males (stage 8) typically 4.25 × 2.25 mm, pre-ovigerous females (stage 7) typically 4.0 × 2.0 mm.

#### Life-history.

A comparison of the life-histories of *Dynamene bidentata* and *Dynamene magnitorata* from two Atlantic coast locations was made by Holdich (1976). Only a limited number of *Dynamene magnitorata* specimens were available but it showed that this species has a similar sequence of seasonal events (see description for *Dynamene bidentata*). However, whereas *Dynamene bidentata* stage 8 males live for two breeding seasons, those of *Dynamene magnitorata* may only live for one.

#### Habitat.

A mid- to lower littoral and shallow sublittoral species, although sometimes recorded from deeper water. Its range occasionally overlaps that of *Dynamene bidentata*. Juveniles are found associated with a wide range of littoral and shallow water algae, particularly *Corallina* sp., *Rhodomenia palmata*, *Chondrus cripspus* and *Gigartina stellata*. Adults have been found in empty tests of *Balanus crenatus*, amongst ascidians, and in channels within sponges (including those associated with eel grass beds). In the Roscoff region (northern France) adults were frequently found within the encrusting sponge, *Halichondria* sp. In the Azores (São Miguel island) adults have been found sublittorally in the empty tests of *Megabalanus azoricus*, as well as intertidally among algae on the islands of Terceira, São Miguel and Santa Maria. On Fuerteventura (Canary Islands) adult males were caught using a surface dip net. In the Chafarinas Islands off Mediterranean Morocco they have been recorded from 0.0m down to 20.0 m on a variety of algae. Like *Dynamene bidentata* (Harvey et al. 1973), *Dynamene magnitorata* adults were found to have a tolerance to high air temperatures, i.e., 38 °C (Holdich 1976). However, survival at 5 °C was much lower for *Dynamene magnitorata* compared to *Dynamene bidentata* (Holdich 1976) and this may be the reason it has not colonized more northerly regions.

#### Colour.

Individuals exhibit a wide variety of colours, often matching the colour of their background, the predominant colours being coralline-pink and brown, rather than the greens and yellows seen in *Dynamene bidentata*. Individuals sometimes exhibit polychromatism caused by white, dorsal, non-adaptable chromatophores, as seen some other species (Holdich 1969, 1976).

#### Geographical distribution.

The distribution of this species shown in Holdich (1970) has been extended by the present study. It has been recorded from southern England (a single specimen only that may be the result of a stranding), the Channel Islands, around the coasts of Brittany, the Atlantic Iberian Peninsula and northwest Africa, the islands of the Azores, Canary Islands and Madeira in the Macaronesian archipelagos, and in the Mediterranean along the European and African coasts, and also Egypt (Fig. 5E).

#### Remarks.

Almost all the *Dynamene* specimens found in the Azores during recent field work belonged to *Dynamene magnitorata*. However, *Dynamene* was less prevalent in the benthic community when comparing with Canaries and Portugal (pers. obs., unpublished data). Maggiore and Fresi (1984) described *Dynamene bidentata* from the Bay of Naples, but in fact examination of the specimen showed it to be a male *Dynamene magnitorata*. If the author’s had compared an actual *Dynamene bidentata* with their specimen then they would have realized this, particular as it is so much smaller than any known *Dynamene bidentata* specimen. *Dynamene magnitorata* has only rarely been recorded in the Mediterranean, i.e. twice in Spain, and once in each of Egypt, Italy, Monaco and Tunisia, although it was found to be common on the Chafarinas Islands off Morocco (Castellanos et al. 2003) (see Suppl. material 1).

### Dynamene tubicauda

Taxon classificationAnimaliaIsopodaSphaeromatidae

Holdich, 1968

#### Restricted synonymy.

*Dynamene tubicauda* Holdich (1968).

*Dynamene tubicauda*: Holdich (1968a, 1970); Lombardo (1984); Borg et al. (2006).

#### Material examined.

Specimens were examined from six Italian locations in the Bay of Naples and off the island of Elba, and one location off Malta - see the Suppl. materials 1 and 2. A number of literature records from Sicily have been included as the diagrams clearly indicate this species (Lombardo 1984).

#### Key morphological characters.

The morphology of this species is unique amongst the known *Dynamene* species - in stage 8 males the pereon length and width are similar; the epimera and front of the head form a shelf; the antennular peduncle is expanded; there are two widely separated, peg-like pleotelsonic bosses; and the pleotelsonic foramen is at the end of a ventrally-closed tube (Fig. 2I). In stage 7 females the body is also flattened with the epimera forming a shelf round the body; the pleotelsonic foramen is at the end of a well-developed tube (Figs 3J–K). See keys to stage 8 males and stage 7 females.

#### Size.

Adult males (stage 8) typically 3.0 × 2.0 mm, pre-ovigerous females (stage 7) typically 2.5 × 2.0 mm.

#### Life-history.

Nothing is known of the life-history of this species, other than the fact that sexual dimorphism occurs with males developing the bidentate process characteristic of the genus. Holdich (1968) only recorded males, but both sexes have been recorded in the present study. Lombardo (1984) was the first to describe the adult female.

#### Habitat.

This species has been found between 2-30 m amongst algae in muddy/sandy and coralline habitats, rock scrapings, freely swimming at 30 m, and also in sea grass meadows (Lombardo 1984, Borg et al. 2006).

#### Colour.

Pale yellow. No polychromatism was observed.

#### Geographical distribution.

The distribution of this species shown in Holdich (1970) has been extended by the present study. However, it appears to be restricted to the eastern Mediterranean, having only been recorded off the west coast of Italy (Holdich 1968), Sicily (Lombardo 1984) and Malta (Borg et al. 2006) (Fig. 5F). The most northerly record is for the island of Elba and the most southerly is off Malta.

#### Remarks.

The distribution of this species is the most restricted of all the *Dynamene* species along the NE Atlantic-Black Sea axis. Considering the large number of samples examined during this study this restricted distribution is most likely real. Its unusual flattened shape and the position of the pleotelsonic foramen at the end of a tube, even in adult males, may be an adaptation to inhabiting sediments.

### Dynamene sp.

Taxon classificationAnimaliaIsopodaSphaeromatidae

#### Material examined.

Two stage 8 males. See the Suppl. materials 1 and 2.

#### Key morphological characters.

The bilobed pleotelsonic boss has a posteriorly-directed spine not seen in any other stage 8 males. The uropodal exopod is wide and the body markedly hirsute.

#### Habitat.

Known only from the stomach contents of a black scorpionfish *Scorpaena porcus*.

#### Geographical distribution.

Known only known from NW Aegean Sea.

#### Remarks.

Only two specimens have been found, both stage 8 males, and both from the stomach contents of a black scorpionfish, *Scorpaena porcus*. This could well be a new species of *Dynamene*, but more material is needed to confirm this. It may even be related to the hirsute specimens found in the Balearic Islands and the Greek island of Chios. The fish is known to be a bottom feeder in the Black Sea, close to where the specimen came from, which was in the NW Aegean, where it occurs at 20–40 m depth (Başçïnar and Saǧlam 2009). Rafrafi-Nouira et al. (2016) examined the diet of *Scorpaena porcus* from waters off the coast of Tunisia, but the only isopods they found were listed as unidentified.

## Discussion

Three species of *Dynamene* occur on the shores of the continent and islands of the NE Atlantic Ocean (*Dynamene bidentata*, *Dynamene magnitorata* and *Dynamene edwardsi*). In recent field work, no *Dynamene* specimens were collected in Scandinavia or Iceland (pers. obs., unpublished data). This is probably due to the fact that members of this genus may not be able to tolerate cold water and weather. For example, studies by Holdich (1968b, c, 1970) were meant to be carried out on the Gower Peninsula in South Wales, but the severe and long-lasting winter of 1962-1963 decimated the populations, as well as those of *Balanus perforatus*, and the study site was relocated to western Pembrokshire in 1964 (SW Wales), where the populations of both were unaffected. Moyse and Nelson-Smith (1964) showed that when sea and air temperatures were below 5 °C for a long period, viable broods were not produced by females of *Dynamene bidentata*. Moreover, with lower average air temperatures, populations of *Dynamene* must restrict their growth phases to fewer months of the year (Holdich 1976). The previously known northerly limit of *Dynamene* was Ardrossan in the west of Scotland (Holdich 1970). In this study, we extended the northern range of this genus to Clatholl in the north of Scotland, and recent surveys by British workers have shown that it also occurs in the Shetland Islands north of Scotland. There are a number of records for the Western Isles off Scotland (Fig. 5A) that are warmed by the Gulf Stream. However, one record is shown from north-eastern England (Fig. 5A), which tends to be colder than the west coast due to a lack of influence from the Gulf Stream, but it is not known if a permanent population exists there. It may represent a stranding from a population elsewhere. Holthuis (1956) recorded *Dynamene bidentata* from the other side of the North Sea in The Netherlands. He was of the opinion that it was not indigenous there, but was occasionally stranded with flotsam and jetsam. There are old records in the literature of *Dynamene bidentata* for eastern Scotland (Scott 1899) and also for south-east England (Butler 1878), but none (other than the record mentioned above) have come to light in the last few decades.

*Dynamene bidentata* is the only species present in the British Isles (Holdich 1969, 1970; Holdich and Lincoln 1974). Although in our databases there is a record of *Dynamene magnitorata* in southern England, we believe this probably does not represent an actual permanently established population. However, *Dynamene magnitorata* is common on Guernsey (Channel Islands), which is not that far geographically from the south of England. According to Holdich (1970), and confirmed by the current study, *Dynamene bidentata* is distributed along the Atlantic coasts of Europe from the northern British Isles to Portugal. Barrois (1888) recorded *Dynamene bidentata* from the Azores, and it is listed as being present there by Ferraz et al. (2004) and Borges et al. (2010). Rodrigues (1990) recorded it as being common on the island of Flores. However, none of the specimens we have examined from the Azores have been of this species, and the records may well have been *Dynamene magnitorata* or *Dynamene edwardsi*. Pereira et al. (2006), Guerra-García et al. (2011), Izquierdo and Guerra-García (2011), Guerra-García et al. (2012) and Torrecilla-Roca and Guerra-García (2012) recorded it from southern Portugal and southwest Spain, and indicated that these regions as the most meridional locations where this species was collected. Our observations extend the distribution of *Dynamene bidentata* further south, i.e., Akhfenir in Morocco and Tenerife and Gran Canaria in the Canary Islands. Because *Dynamene bidentata* can survive at temperatures up to 38 °C (Harvey et al. 1973), it is possible that this species occurs further south.

During the current study the authors examined many collections from the Mediterranean and we did not find any *Dynamene bidentata*. It has been pointed out above that Torelli’s (1930) ‘*Dynamene bidentata*’ from the Bay of Naples is in fact *Dynamene bicolor*, as are a number of other references to *Dynamene bidentata* in the literature. Also, Maggiore and Fresi’s (1984) ‘*Dynamene bidentata*’ from the Bay of Naples is a *Dynamene magnitorata*. From the examination of some other collections we also conclude that Castello’s (1986) ‘*Dynamene bidentata*’ is an *Ischyromene* sp., that Kirkim’s (1998) ‘*Dynamene bidentata*’ is *Dynamene bicolor*, and that Castellanos’ et al. (2003) ‘*Dynamene bidentata*’ is *Dynamene magnitorata*. It is not impossible that *Dynamene bidentata* occurs in the western Mediterranean as it has been recorded close to the Strait of Gibraltar (Torrecilla-Roca and Guerra-García 2012), but currently there is no evidence for this.

On Atlantic mainland coasts and islands, *Dynamene bidentata*, *Dynamene edwardsi* and *Dynamene magnitorata* are usually present in the midlittoral to sublittoral zones, although occasionally they are found higher up the shore. Usually the juveniles are present among the fronds of brown, red and sometimes green algae, whilst the adults inhabit cryptic habitats such as crevices, empty barnacle tests, mussel beds and encrusting organisms. Individuals often match the colour of the algae they are feeding on and additional camouflage is afforded by linear and globular patterns of white chromatophores on the dorsal surface (Tinturier-Hamelin 1962, Holdich 1969, 1976). In the Mediterranean and Black Seas, *Dynamene magnitorata*, *Dynamene bifida*, *Dynamene bicolor* and *Dynamene edwardsi* usually inhabit shallow water zones, although the last two species can also be present in deeper water off steep-sided islands. Juveniles of these species inhabit algae whilst adults are usually found in more cryptic habitats, but sometimes amongst algae. *Dynamene tubicauda* has been found between 2-30 metres amongst algae in muddy/sandy and coralline habitats, rock scrapings, freely swimming at 30 m, and also in sea grass meadows (Lombardo 1984, Borg et al. 2006, Holdich, pers. obs.). The vertical range of *Dynamene bicolor* is the largest, extending from shallow-water algae and cryptic habitats such as barnacles down to 33 m off steep-sided islands. The vertical ranges of some *Dynamene* species may overlap, e.g., *Dynamene bidentata* and *Dynamene magnitorata* on Atlantic Ocean shores, although the latter usually occurs at a lower level on the shore (Holdich 1970, Arrontes and Anadón 1990a; Castelló and Carballo 2001, Guerra-García et al. 2011, Izquierdo and Guerra-García 2011). *Dynamene bicolor* and *Dynamene edwardsi* frequently inhabit the same shallow-water algae in the Mediterranean.

## Conclusions

Six species of *Dynamene* are present along the NE Atlantic-Black Sea axis, and one species extends into the Red Sea. It would appear that *Dynamene bidentata* is restricted to coastal habitats of the NE Atlantic, no evidence was found to suggest it inhabits the Mediterranean. *Dynamene magnitorata* has a wider geographical range, occurring on coastal habitats of the NE Atlantic as well as those of the Mediterranean. *Dynamene edwardsi* has the widest geographical range of the six species under consideration, extending from the Macaronesian archipelagos in the NE Atlantic, down the north-western coast of Africa, through the Mediterranean into the Suez Canal and Red Sea. It is not known if a recent record from South Africa represents an introduction or an established population. *Dynamene bicolor*, *Dynamene bifida* and *Dynamene tubicauda* are restricted to the Mediterranean, although *Dynamene bicolor* also extends into the Black Sea. *Dynamene bicolor* is the most commonly found and most wide-spread *Dynamene* species in the Mediterranean. *Dynamene bifida* has only been recorded at six locations, but its range extends from southern Spain to Turkey. *Dynamene tubicauda* has the smallest geographical range having only been recorded for Italy and Malta. Some species have large vertical ranges, having been found intertidally down to 30 m. It is highly probable that some of the records for the *Dynamene* species are the result of introductions via fouling organisms attached to ocean-going vessels, e.g., *Dynamene magnitorata* and *Dynamene bifida* with their sporadic distribution in the Mediterranean, and *Dynamene edwardsi* in South Africa.

There are still a number of outstanding issues relating to *Dynamene* that can only be dealt with if more material becomes available. Firstly, the status of the hirsute species from the Balearic Islands and the Greek island of Chios – are these a form of *Dynamene edwardsi* or a new species? Secondly, the status of ‘*Dynamene torelliae*’ – is it really synonymous with *Dynamene bicolor* from the Black Sea? Thirdly, the status of the specimens found in the *Scorpaena porcus* stomach, which appears different from the other species, but cannot be confirmed until more stage 8 males are found. Fourthly, a genetic analysis of all the species needs to be carried out to ascertain the taxonomic status and species boundaries, and the phylogenetic relationships between species, especially those in the Mediterranean and Black Seas. Currently, only *Dynamene bidentata*, *Dynamene magnitorata* and *Dynamene edwardsi* from NE Atlantic coasts have been analyzed, and have been found to be distinct.

### Key to the adult males (stage 8) of *Dynamene* spp. along the NE Atlantic-Black Sea axis

**Table:** 

1	With a bidentate process arising from posterior margin of pereonite 6 - sub-adult and adult ♂ *Dynamene* (Figs 1, 2, 3)	**2**
–	Without bidentate arising from posterior margin of pereonite 6	**juvenile and ♀ *Dynamene*** (see key to females)
2	With large bidentate process arising from posterior margin of pereonite 6: adult ♂ *Dynamene* (Figs 1D, 2A–K)	**3**
–	With small or medium bidentate process arising from posterior margin of pereonite 6	**sub-adult ♂ *Dynamene*** (Fig. 4–lower row 6-7)
3	Pereon length and width similar; epimera and front of head forming a shelf; antennular peduncle expanded; two widely separated, peg-like pleotelsonic bosses; pleotelsonic foramen at end of a ventrally-closed tube (Fig. 2I)	***Dynamene tubicauda***
–	Pereon length greater than width, pleura and front of head not forming a shelf; antennular peduncle not expanded; pleotelsonic boss single	**4**
4	Bidentate processes large, tapering and with a well-developed, downwardly-directed accessory process a quarter of the way from the apex; pleotelsonic boss very small with raised pointed corners (Fig. 2G–H)	***Dynamene bifida***
–	Bidentate processes without well-developed accessory process; pleotelsonic boss well-developed, without raised pointed corners	**5**
5	Apices of bidentate processes swollen, each with short, downwardly-directed spur; pleotelsonic boss plate-like with two forward-facing pegs; body exhibiting various degree of setation, sometimes hirsute (Fig. 2E–F)	***Dynamene edwardsi***
–	Bidentate processes without swollen apices or spurs, pleotelsonic boss not plate-like	**6**
6	Pleotelsonic boss comprised of two right-angled triangular structures separated by a deep groove (however, the boss may be very low lying in some specimens, e.g. those from the Black Sea); arms of bidentate process tapering to point, rugose dorsally (Fig. 2J–K)	***Dynamene bicolor***
–	Pleotelsonic boss comprising two hemispherical structures separated by a wide or a narrow groove, joined at the base	**7**
7	Pleotelsonic boss large, bilobed, two halves separated by a narrow groove; arms of bidentate process of similar width with along length, dorsally tuberculate (Fig. 2C–D)	***Dynamene magnitorata***
–	Pleotelsonic boss large, bilobed, two halves separated by a wide v-shaped groove; arms of bidentate process tapering to point, sparsely rugose dorsally (Fig. 2A–B)	***Dynamene bidentata***

### Key to pre-ovigerous females (stage 7) and juveniles of *Dynamene* spp. along the NE Atlantic-Black Sea axis

**Table:** 

1	Sphaeromatid without process arising from the posterior margin of the pereonite 6, and with simple pleotelsonic foramen; with or without dorsal tuberculation	**juvenile and ♀♀ *Campecopea* , *Dynamene* and *Ischyromene***
–	Without tuberculation on surface of posterior pereonites, pleonites and/or pleotelsonic dome (Figs 1A, B; Figs 4-upper row 6-8, 3A–M)	juvenile and ♀♀ *Dynamene*...**2**
2	Body flattened, epimera flattened to form a shelf round the body; pleotelsonic foramen at end of a well-developed tube (Fig. 3J-K)	***Dynamene tubicauda***
–	Body convex, pleura not flattened to form shelf round body; pleotelsonic foramen either flush with edge of pleotelson or at end of a short tube	**3**
3	Pleotelsonic dome smoothly rounded in side view, pleotelsonic foramen open and flush with edge of pleotelson or at end of short tube	**4**
–	Pleotelsonic dome keeled in side view, with or without a median protuberance	**5**
4	Pleotelsonic foramen open and flush with edge of pleotelson (Fig. 3A–B)	***Dynamene bidentata***
–	Pleotelsonic foramen at end of short tube (Fig. 3L–M)	***Dynamene bifida***
5	Pleotelsonic dome keeled in side view, pleotelsonic foramen flush with edge of pleotelson	Fig. 3C–D – ***Dynamene magnitorata*** and Fig. 3H, I – ***Dynamene bicolor***
–	Pleotelsonic dome keeled in side view, with median protuberance; pleotelsonic foramen at end of short tube (Fig. 3E, F, G)	***Dynamene edwardsi***

**Notes**: When identifying *Dynamene* juveniles and ♀♀ care must be taken not to confuse them with those of *Ischyromene lacazei* Racovitza, 1908 and *Campecopea lusitanica* (Nolting, Reboreda & Wägele, 2008). If in doubt, then consult Schüller and Wägele (2005) and Bruce and Holdich (2002) respectively.

Except for size, juveniles are very similar to stage 7 females. *Dynamene magnitorata* and *Dynamene bicolor* females are very similar and cannot be keyed out, except on size – on average *Dynamene magnitorata* tends to be larger (see main text). Ovigerous females are very similar between species and it is not possible to create a key for them. They are characterized by metamorphosed mouthparts, ventral marsupium, wide body and a pleotelsonic foramen that is more upturned and which gradually becomes closed posteriorly (Fig. 4–upper row 8).

## Supplementary Material

XML Treatment for Dynamene

XML Treatment for Dynamene bidentata

XML Treatment for Dynamene bicolor

XML Treatment for Dynamene bifida

XML Treatment for Dynamene edwardsi

XML Treatment for Dynamene magnitorata

XML Treatment for Dynamene tubicauda

XML Treatment for Dynamene sp.

## Supplementary materials

Supplementary material 1 Material examined in this study Data type: occurence Explanation note: Each entry shows the number of specimens observed for each life history stage, habitat, person who provided the specimen(s), location and country, co-ordinates and other information. This dataset is made available under the Open Database License (http://opendatacommons.org/licenses/odbl/1.0/). The Open Database License (ODbL) is a license agreement intended to allow users to freely share, modify, and use this Dataset while maintaining this same freedom for others, provided that the original source and author(s) are credited.Pedro E. Vieira, Henrique Queiroga, Filipe O. Costa, David M. Holdich

Supplementary material 2 List of locations where *Dynamene* specimens were recorded in this study Data type: occurence Explanation note: Complete list of locations and respective regions and co-ordinates where *Dynamene* specimens were recorded in this study, organized by species. This dataset is made available under the Open Database License (http://opendatacommons.org/licenses/odbl/1.0/). The Open Database License (ODbL) is a license agreement intended to allow users to freely share, modify, and use this Dataset while maintaining this same freedom for others, provided that the original source and author(s) are credited.Pedro E. Vieira, Henrique Queiroga, Filipe O. Costa, David M. Holdich
