# Supplementary material for: Distribution and species identification in the crustacean isopod genus Dynamene Leach, 1814 along the North East Atlantic-Black Sea axis
Source: Zookeys. 2016 Nov 23;(635):1–29. doi: 10.3897/zookeys.635.10240 (PMC5126507; doi:10.3897/zookeys.635.10240)
Supplement: Supplementary material 1 — Material examined in this study [file zookeys-635-001-s001.docx]

**Material examined**

***Dynamene bicolor* (Rathke, 1837)**

**Mediterranean**

**Spain**

8 stage 8 ♂♂, 1 stage 7 ♂, 4 ♀♀, 2 juvs; 0-1 m, H. Zibrowius (Endoume Marine Lab.), 1986; Alicante, 38⁰ 20’ N, 0⁰ 29’ E (RMNH.CRUS.1.7573).

2 stage 8 ♂♂ (largest 3 mm x 1.5 mm) 2 stage 7 ♀♀ (largest 3 mm x 1.5 mm); 0.1 m, L. B. Holthuis, 1954; Rosen by Casa Zariguiey (Baai van Cadaques), 42⁰ 14’ N, 3⁰ 12’ E (RMNH.CRUS.1.7493).

3 stage 8 ♂♂, 3 ♀♀; amongst barnacles, shallow water, D. M. Holdich, 1984; Arenal d’en Castell, Menorca, 40⁰ 1’ N, 4⁰ 10’ E (RMNH.CRUS.1.7575).

1 stage 7 ♂, 4 ♀♀; red and brown algae in rock pool amongst the encrusting red algae, D. M. Holdich, 1984; Arenal d’en Castell, Menorca, 40⁰ 1’ N, 4⁰ 10’ E (RMNH.CRUS.1.7576).

1 stage 8 ♀; no habitat details, J. Castello (University of Barcelona, Spain), 1983; Cap d’Artutx, Spain, 39⁰ 55’ N, 3⁰ 49’ E (RMNH.CRUS.1.7578).

1 ♀: no habitat details, J. Castello (University of Barcelona), 1983; Cala Morell, Menorca, 40⁰ 3’ N, 3⁰ 53’ E (RMNH.CRUS.1.7579).

2 juvs; no habitat details, J. Castello (University of Barcelona), 1983; Botafoc, Ibiza, 38⁰ 54’ N, 3⁰ 49’ E (RMNH.CRUS.1.7580).

3 ♀♀; shallow-water encrusting red algae and *Padina*, D. M. Holdich, 1978; Calla Longa, Ibiza, 38⁰ 57’ N, 1⁰ 31’ E (RMNH.CRUS.1.7582).

2 ♀♀; shallow-water algae, D. M. Holdich, 1978; San Antonio, Ibiza, 38⁰ 58’ N, 1⁰ 18’ E (RMNH.CRUS.1.7583).

1 ♀; no habitat details, J. Castello (University of Barcelona), 1984; Estany d’es Peix, Formentera, 38⁰ 43’ N, 1⁰ 25’ E (RMNH.CRUS.1.7584).

**France**

13 stage 8 ♂♂, 9 stage 7 ♀♀, 3 stage 8 ♀♀, 6 juvs; 5-6 m, H. Zibrowius (Endoume Marine Lab.), 1980s; Ponteau, nr Marseille, 43⁰ 22’ N, 5⁰ 76’ E, and Marseille, 43⁰ 17’ N, 5⁰ 22’ E (RMNH.CRUS.1.7586).

1 stage 8 ♂, 2 ♀♀; littoral, H. Schmalfuss (Germany), 1962; Villefranche-sur-Mer, 43⁰ 42’ N, 7⁰ 18’ E (RMNH.CRUS.1.7588).

Many ♀♀ and juvs; shallow-water algae, M-L. Roman (University of Marseille), 1978; Lagune du Brusc (near Toulon to the east of Marseille), 43⁰ 04’ N, 5⁰ 48’ E (RMNH.CRUS.1.7589).

1stage 8 ♂, 1 stage 7 ♂, 1 stage 8 ♀; amongst algae at 1m depth; Argelès, 42⁰ 32’ N, 3⁰ 1’ E. No other details. (RMNH.CRUS.1.7495).

15 stage 8 ♂♂, 5 stage7 ♀♀, 2 stage 8 ♀♀; some from 12-15 m, others from rocky face at N. Citadel, H. Zibrowius, pre-1987; NW Corsica (Calvi), 42 ⁰ 34’ N, 8⁰ 45’ E. Pre-1987, but some samples dated from 1950s - these are from previous workers (e.g. Pérès and Picard at Endoume Marine Station). (RMNH.CRUS.1.7585).

1 stage 8 ♂ (4 mm), 1 ♀; there are 4 stage 8 ♀♀, but it is difficult to tell which species they are as both *D. bifida* and *D. torelliae* were found together; rocky shore, between seaweed, 0-1 m, 1957; Banyuls-sur-Mer, 42 ⁰ 29’ N, 3⁰ 7’ E (RMNH.CRUS.1.7498).

**Italy**

59+ stage 8 ♂♂, 32+ stage 7 ♂♂, 100+ ♀♀, 100+ juvs; shallow water *Cystoseira* and *Sargassum* and crevices, D. M. Holdich, 1967; Mergellina Harbour, Bay of Naples, 40⁰ 49' N, 14⁰ 13' E (RMNH.CRUS.1.7527 and RMNH.CRUS.1.7591).

1 stage 7 ♀; J. A. W. Lucas, 1953; Posillipo, Bay of Naples, 40⁰ 48’ N, 14⁰ 12’ E (RMNH.CRUS.1.7501).

1 stage 7 ♂; amongst algae near Zool. Station, Bay of Naples, L. B. Holthuis, 1950; 40⁰ 49’ N, 14⁰ 13’ E (RMNH.CRUS.1.7502).

4 stage 8 ♂♂, 1 stage 7 ♂; amongst mussels, JAW Lucas. 1953; Via Caracciola (Grand Hotel), Bay of Naples, 40⁰ 50’ N, 14⁰ 13’ E (RMNH.CRUS.1.7503).

1 stage 8 ♂ (damaged); amongst barnacles, J. A. W. Lucas, 1953; Rodonde, Gulf of Naples, 40⁰ 49’ N, 14⁰ 13’ E (RMNH.CRUS.1.7504).

1 stage 7 ♀; amongst barnacles, J. A. W. Lucas, 1953; Rodonde, Bay of Naples, 40⁰ 49’ N, 14⁰ 13’ E (RMNH.CRUS.1.7505).

1 stage 8 ♂; amongst barnacles; J. A. W. Lucas, 1953; Mergellina, Bay of Naples, 40⁰ 49’ N, 14⁰ 13’ E (RMNH.CRUS.1.7506).

3 stage 8 ♂♂, 1 ♀, 1 juv; no habitat details, H. Zibrowius, 1980s; Elba island, 42⁰ 48’ N, 10⁰ 08’ E (RMNH.CRUS.1.7599).

1 stage 8 ♂; 3 stage 7 ♂♂, 1 stage 8 ♀, 2 ♀♀; no habitat details, G. Messana, 1990s; St Andrea, Elba island, 42⁰ 48’ N, 10⁰ 8’ E. Specimens returned to collector.

6 stage 8 ♂♂, 1 stage 8 ♀, 8 ♀♀; no habitat details, H. Zibrowius, 1980s; Isola di Bergegge, Gulf of Genova ,44⁰ 14’ N, 8⁰ 26’ E (RMNH.CRUS.1.7600).

16 males stage 8 ♂♂, 3 stage 7 ♂♂, 6 ♀♀ females and juvs; 3.5 m on algae, U. Schieke and E. Fresi, 1968, 1970; E.La Voru au Wand, Ms Mary, S. Angelo, Ischia island, Bay of Naples, 40⁰ 44’ N, 13⁰ 57’ E (RMNH.CRUS.1.7605).

10 male 8 ♂♂, 8 stage 7 ♂♂, 13 ♀♀, 3 juvs; *Cystoseira* at 4 m (S. Anna), *Hydroides* and algae at 1-3 m, algae and detritus at 35 m (P. S. Pancrazio), algae and sediment at 8-12 m (L’Ameno), U. Schieke and E. Fresi, 1968, 1969; S. Anna, L’Ameno, P.S. Pancrzio, Ischia island, Bay of Naples, 40⁰ 44’ N, 13⁰ 57’ E (RMNH.CRUS.1.7606).

2 stage 8 ♂♂, 2 stage 7 ♂♂, 2 stage 8 ♀♀, 49 ♀♀ and juvs; 3.5 m detritus, *Dictyoptera* by laboratory, 0.5 m (Castello), U. Schieke and E. Fresi, 1968, 1969; Castello and Ecological Laboratory, Ischia island, Bay of Naples, 40⁰ 44’ N, 13⁰ 57’ E (RMNH.CRUS.1.7607)

16 stage 8 ♂♂, 15stage 6/7 ♂♂, 29 ♀♀ and juvs; 1-4 m on algae below lab., U. Schieke and E. Fresi, 1968; Ecological Laboratory, Ischia island, Bay of Naples, 40⁰ 44’ N, 13⁰ 57’ E (RMNH.CRUS.1.7608).

5 stage 8 ♂♂, 4 stage 7 ♂♂, 30 ♀♀ and juvs; 0.5-4 m on *Cystoseira* and *Dictyoptera*, U. Schieke and E. Fresi, 1968; Castello and below Ecological Laboratory, Ischia island, Bay of Naples, 40⁰ 44’ N, 13⁰ 57’ E (RMNH.CRUS.1.7609).

3 stage 8 ♂♂; trottoir, U. Schieke and E. Fresi, 1968; Palermo, Sicily, 38⁰ 11’ N, 13⁰ 20’ E (RMNH.CRUS.1.7601).

**Croatia**

1 stage 7 ♂; no other details, 1960; Rovinji, 45⁰ 4’ N, 13⁰ 38’ E, (RMNH.CRUS.1.7507) (labelled as from Yugoslavia).

**Greece**

3 stage 8 ♂♂,1 stage 7 ♂, 1 stage 8 ♀, 1 ♀; shallow water algae, D. A. Jones, 1967; Emborios Bay, Chios island, 38⁰ 11’ N, 26⁰ 1’ E (RMNH.CRUS.1.7529).

11 stage 8 ♂♂ (averaging 3 mm in length), 1 stage 7 ♂, 2 stage 8 ♀♀, 9 ♀♀ and juvs,; variety of algae, e.g. *Cystoseira*, *Sargassum* at a variety of depths, e.g 0.5 m down to 33 m, D. A. Jones, 1967; Cathedral Rock, Emborios Bay, Cape Maskla, Chios island, 38⁰ 11’ N, 26⁰ 1’ E (RMNH.CRUS.1.7645).

7 stage 8 ♂♂, 2 stage 8 ♀♀; from oscula of sublittoral sponge, D. M. Holdich, 1980; Gerakini Beach, Halkidiki Peninsula, 40⁰ 16’ N, 23⁰ 26’ E (RMNH.CRUS.1.7612).

2 stage 8 ♂♂; from sublitoral coralline algae, D. M. Holdich, 1980; Gerakini Beach, Halkidiki Peninsula, 40⁰ 16’ N, 23⁰ 26’ E (RMNH.CRUS.1.7613).

22 stage 8 ♂♂, 4 stage 8 ♀♀, 1 ♀, (plus 1 male *Campecopea hirsuta*); from surface of empty *Pinna* shell at 2 m, D. M. Holdich, 1980; Gerakini Beach, Halkidiki Peninsula, 40⁰ 16’ N, 23⁰ 26’ E (RMNH.CRUS.1.7614).

4 juvs; from fucoid algae at 0.5 m, D. M. Holdich, 1980; Ouranoupolis, Halkidiki Peninsula, 40⁰ 19’ N, 23⁰ 58’ E (RMNH.CRUS.1.7615).

1 stage 8 ♂ (damaged). 0-15 m, W. J Wolffen and M. Loosies, 1967; Missolonghi (to west of the Gulf of Corinth, now called Mesolongion, 38⁰ 22’ N, 21⁰ 25’ E (RMNH.CRUS.1.7508).

9 stage 7 ♀♀; no habitat details, W. J. Wolff and M. Loosjes, 1964; Sulaora, Epirus (bordering Albania), 39⁰ 30’ N, 20⁰ 15’ E (RMNH.CRUS.1.7509).

1 stage 7 ♂, 1 ♀; weed under stones, <1 m, D. A. Jones, 1967; Corfu island, 39⁰ 36’ N, 19⁰ 49’ E (RMNH.CRUS.1.7643).

1 ♀; encrusting red seaweeds, D. M. Holdich, 1983; Matala, Crete island, 34⁰ 59’ N, 24⁰ 44’ E (RMNH.CRUS.1.7644).

**Turkey**

2 stage 8 ♂♂ (4.2 x 2 mm), 1 stage 7 ♂; intertidal and shallow water algae and other habitats, F. Kirkim, mid-1990s; Izmir region, 38⁰ 28' N, 27⁰ 6' E (RMNH.CRUS.1.7657).

10 stage 8 ♂♂, 5 stage 7 ♂♂, 17 stage 7 ♀♀, plus 1 *Cymodoce* ♀ sp; 0-50 cm deep, C. Swennen, 1957; Antalya, 36⁰ 53' N, 30⁰ 44' E (RMNH.CRUS.1.7510).

25 stage 8 ♂♂, 2 stage 7 ♂♂, 5 stage 8♀♀, 13 stage 7 ♀♀ and juvs; 5-20 cm deep, C. Swennen, 1959; Antalya harbour, 36⁰ 53' N, 30⁰ 42' E (RMNH.CRUS.1.7511).

1 stage 8 ♀; C. Swennen, 1959; Antalya harbour, 36⁰ 53' N, 30⁰ 42' E (RMNH.CRUS.1.7512).

**Israel**

2 stage 8 ♂♂, 1 stage 7 ♂, 1 ♀, 1 juv; algae from rocky shore; T. Haran (Tel Aviv University), 1977-78; Mikhmoret (south of Haifa), 32⁰ 24' N, 34⁰ 52' E (RMNH.CRUS.1.7528).

9 stage 8 ♂♂ (largest 4.0 x 2.0 mm), 14 stage 6/7 ♂♂ (3.25 x 1.5 mm), 60 ♀♀ (largest 3.5 x 1.4 mm), 63 juvs; algae on rocky shore (*Ulva*, *Sargassum*, *Jania*, *Acanthophora*, *Cystoseira*), T. Haran (Tel Aviv University), 1977-78; Mikhmoret (south of Haifa), 32⁰ 24' N, 34⁰ 52' E (RMNH.CRUS.1.7649).

1 stage 6 ♂, 9 ♀♀, 4 juvs; littoral algae, L. Fischelson, 1976; Rosh HaNikva, north of Haifa, 33⁰ 5' N, 35⁰ 6' E (for Haifa) (RMNH.CRUS.1.7650).

7 stage 8 ♂♂, 4 stage 7 ♂♂, 6 ♀♀; littoral algae (*Cystoseira*, *Laurencia*, *Jania*), L. Fischelson, 1976; Acre, north of Haifa, 32⁰ 55' N, 35⁰ 4' E (for Haifa) (RMNH.CRUS.1.7652).

3 stage 7 ♂♂, 1 stage 6 ♂, 7 ♀♀, 7 juvs; littoral algae (*Pterocladia*, *Acanthophora*, *Ulva*, *Jania*, *Bryopsis*, *Sargassum*, *Padina*), L. Fischelson, 1976; Bat-Yam, south of Tel Aviv, 32⁰ 5 N', 34⁰ 48' E (for Tel Aviv) (RMNH.CRUS.1.7654).

4 ♀♀, 4 juvs; littoral algae (*Pterocladia*, *Colpomenia*), L. Fischelson, 1977; Michmoreth north of Netania, 32⁰ 20' N, 34⁰ 52' E (for Netania) (RMNH.CRUS.1.7655).

2 ♀♀, 4 juvs; littoral algae (*Jania*, *Centroceras*), L. Fischelson, 1977; Palmhim, north of Ashdod, 31⁰ 55' N, 34⁰ 42' E (RMNH.CRUS.1.7656).

**Malta**

1 stage 7 ♂, 1 ♀; intertidal rock pool, D. M. Holdich, 1997; Mellieha Bay, 35⁰ 58’ N, 14⁰ 21’ E (RMNH.CRUS.1.7661).

3 stage 8 ♂♂, 3 stage 7 ♀♀; rocky sea bed, 1-2 m, S. Schembri, 2010; Cirkewwa, 35⁰ 59’ N, 14⁰ 19’ E (RMNH.CRUS.1.7662).

**Egypt**

2 stage 8 ♂♂; M. M. Atta, 1981; Agamy area, Alexandria, 31⁰ 9’ N, 29⁰ 55’ E (RMNH.CRUS.1.7667).

**Tunisia**

4 stage 8 ♂♂; Th Monod; Tunis (?), 38⁰ 48’ N, 10⁰ 10’ E. Museum National d’Histoire Naturelle, Paris collection. IS 683.

**Black Sea**

**Turkey**

1 stage 7 ♂, 1 stage 8 ♀♀, , 1 ♀, 1 juv; no habitat details, A. M. Gozler, 2007; Rýze coast, 41⁰ 01' N, 40⁰ 28' E (RMNH.CRUS.1.7530).

1 stage 8 ♂ (3 x 1.3 mm – very small), 5 stage 7 ♂♂ (3.5 x 1.5), 8 ♀♀ (3.5 x 1.5 mm), 1 juv; no habitat details, A. M. Gozler, 2007; Rýze coast, 41⁰ 01' N, 40⁰ 28'E (RMNH.CRUS.1.7670).

1 stage 8 ♂, 1 stage 6 ♂, 2 stage 8 ♀♀, 4 ♀♀, 13 juvs; infralittoral down to 5 m depth, algae (*Cystoseira* spp.), amongst *Mytilus galloproincialis*, G. Gönlügür, 2006; Sinop coast, 42⁰ 00' N, 35⁰ 10' E (RMNH.CRUS.1.7671).

1 stage 7 ♀; 0-5m deep. C. Swennen, 1969; Trabzon harbour (north coast of Turkey near eastern end of Black Sea), 40⁰ 57' N, 39⁰ 43' E (RMNH.CRUS.1.7514).

**Romania**

1 stage 8 ♂,1 stage 6 ♂, 1 stage 8 ♀, 5 ♀♀, 6 juvs; hard bottoms and silt amongst *Mytilus* and empty shells of *Balanus improvisus* at 5-10 m, M. Băcescu, 1961; eastern Constanta, 44⁰ 12' N, 28⁰ 38' E (RMNH.CRUS.1.7672).

**Bulgaria**

1 stage 8 ♂ (damaged back end), 2 ♀♀ (one damaged); no habitat details, O. G. Kussakin, 1950s; Varna, 43⁰ 13' N, 28⁰ 00' E (RMNH.CRUS.1.7673).

3 stage 8 ♂♂, 1 stage 6 ♂, 2 stage 8 ♀♀, 3 ♀♀, 2 juvs; no habitat details, O. G. Kussakin; label just says Black Sea, could be Varna, 43⁰ 13' N, 28⁰ 00' E (RMNH.CRUS.1.7674).

**Georgia**

1 stage 8 ♂; 0.5-1.5 m, O. G. Kussakin, 1900s; Sukhumi Bay - 43⁰ 00' 12'' N, 41⁰ 00' 55'' E (RMNH.CRUS.1.7675).

***Dynamene bidentata* (Adams, 1800)**

**Atlantic**

**Canary Islands (Spain)**

1 stage 7 ♂, 2 ♀♀, one juv; algae in upper shore pool, D.M. Holdich, 1970; El Medano, Tenerife, 28⁰ 2’ N, 16⁰ 32’ W (RMNH.CRUS.1.7558).

1 juv; intertidal rocky shores among algae during low tide, P. Vieira, 2011; Bañaderos, Gran Canaria, 28° 8' N, 15° 32' W.

**England** (including the Isle of Man)

6 stage 8 ♂♂ (second year, with growth of algae on pleotelson and uropods), 4 stage 7 ♀♀, 5 stage 8 ♀♀ (one with epicarid parasite *Ancyroniscus bonnieri*),; empty *Balanus perforatus* tests, D. M. Holdich, 1996; Trevone Bay, Cornwall, 50⁰ 32’ N, 4⁰ 58’ W (RMNH.CRUS.1.7546 ).

In addition, specimens of *D. bidentata* were examined by DMH from the following locations during the period 1964-1975 (Holdich 1970, 1974, 1976) and subsequently: Bovisand (50°19' 60" N, 04° 06' 60" W); Cape Cornwall (50°07' 01" N, 05° 42' 04" W); Castle Rocks, Falmouth (50° 08' 38" N, 05° 03' 40" W); Derby Haven, Isle of Man (54° 04' 00" N, 04° 37' 00" W); Goodrington Sands (50° 24' 60" N, 03° 33' 60" W); Helford Passage (50° 05' 34" N, 05° 06' 07" W); Ilfracombe (51° 12' 32" N, 04° 07' 46" W); Kennack Sands (50° 00' 00" N, 05° 10' 00" W); Lizard (49° 57' 43" N, 05° 11' 54" W); Marazion (50° 07' 00" N, 05° 27' 00" W); Mullion Cove (49° 59' 49" N, 05° 15' 18" W); Periglio Bay, St Agnes, Scilly Isles (49° 53' 60" N, 06° 24' 00" W); Port Erin, Isle of Man (54° 05' 03" N, 04° 45' 39" W); Portland Harbour (50° 34' 00" N, 02° 26' 00" W); South Milton Sands (50° 15' 00" N, 03° 50' 60" W); St Agnes (50° 19' 01" N, 05° 14' 02" W); St Marys, Isles of Scilly (49° 55' 15" N, 06° 18' 48" W); St Ives (50° 11' 60" N, 05° 28' 00" W); Treyarnon (50° 31' 12" N, 05° 01' 44" W); Wembury, Church Reef (50° 17' 26" N, 04° 46' 46" W); Widermouth (50° 7' 13" N, 04° 33' 37" W); Whitesand Bay (50° 19' 60" N, 04° 15' 60" W).

British Natural History Museum (BMNH) collection:

Port St Mary, Isle of Man (54° 04' 33" N, 04° 44' 21" W)**;** Kimmerridge Bay (50° 36' 39" N, 02° 06' 59" W); Brandy Bay (50° 36' 47" N, 02° 09' 26" W); Hobarrow Bay (50° 36' 33" N, 02° 08' 55" W); St Agnus, Scilly Isles – in sponges and worm tubes (49° 53' 35" N, 06° 20' 25" W); Porth Warne, St Agnus, Scilly Isles - amongst red algae (49° 54' 58" N, 06° 17' 53" W); Porth Hellick, Scilly Isles (49° 55' 07" N, 06° 16' 49" W), Hells Gate Beach, Lundy – intertidal weed (51° 10' 41" N, 04° 20' 02" W).

National Biodiversity Network:

14 records have been used from the NBN database, but details are not given as they can be found on the NBN website: www.searchnbn.net.

ERICA database:

St Martins, Isles of Scilly (49° 58' 48" N, 06° 17' 28" W); Tresco, Isles of Scilly (49° 56' 47"N 06° 20' 18" W); Bryher, Isles of Scilly (49° 57' 08" N, 06° 21' 54" W); Smith Sound, Isles of Scilly (49° 52' 52" N, 06° 21' 59" W); Old Town, Isles of Scilly (49° 54' 09" N, 06° 18' 13" W); Mevagissey (50° 15' 16" N, 04° 46' 01" W); St Mawes (50° 09' 18" N, 05° 01' 20" W); Porthleven (50° 04' 57" N, 05° 19' 21" W); Clovelly (50° 59' 00" N, 04° 23' 00" W), Feock (50° 11' 36" N, 05° 03' 35" W); Gwithian (50° 13' 42" N, 05° 23' 39" W); Mounts Bay (50° 05' 35" N, 05° 22' 27" W); Padstow Bay (50° 34' 49" N, 04° 55' 20" W); Penzance (50° 07' 00" N, 05° 31' 60" W); Portlooe (50° 20' 31" N, 04° 27' 38" W); Portscatho (50° 11' 06" N, 04° 58' 15" W); Rosenithon (50° 02' 48" N, 05° 04' 02" W); Treen (50° 11' 10" N, 05° 36' 05" W); Trevelgue (50° 25' 33" N, 05° 03' 13" W); Weymouth (50° 36' 17" N, 02° 32' 12" W).

**Northern Ireland**

National Biodiversity Network:

22 records have been used from the NBN database, but details are not given as they can be found on the NBN website: www.searchnbn.net.

**Southern Ireland (Eire)**

1 stage 7 ♂, 2 stage 8 ♂♂, two ♀♀, 2 juvs; rocky shore, D. McGrath, 1975; Galway, Southern Ireland (Eire), 53⁰ 16’ N, 9⁰ 3’ W (RMNH.CRUS.1.7519).

1 stage 7 ♂, 8 stage 8 ♂♂, 76 ♀♀ and juvs; rocky shore, D. McGrath, 1975; Galway, Southern Ireland (Eire), 53⁰ 16’ N, 9⁰ 3’ W (RMNH.CRUS.1.7550).

British Natural History Museum (BMNH) collection:

Roundstone (53° 23' 36" N, 09° 51' 27" W), Tory Island (55° 15' 55" N, 08° 13' 49" W), Valentia (51° 53' 59" N, 10° 20' 00" W).

National Biodiversity Data Centre (Ireland):

Ballycotton (51° 49' 59" N, 08° 01' 03" W), Carrownedin (54° 13' 55" N, 09° 05' 21" W), Corkagh Beg (54° 16' 11" N, 08° 45' 17" W), Doonbeg (52° 44' 43" N, 09° 31' 26" W), Dungarvan (52° 44' 43" N, 07° 32' 45" W), Fenit (52° 17' 07" N, 09° 52' 34" W), Finvarra (53° 08' 58" N, 09° 08' 22" W), Garrywilliam (52° 18' 34" N, 10° 03' 17" W), Glengariff (51° 44' 52" N, 09° 32' 56" W), Liscannor (52° 56' 10" N, 09° 26' 16" W), Loughshinny (53° 33' 39" N, 05° 58' 55" W), Mullagh (52° 47' 31" N, 09° 29' 06" W), Rathlee (54° 16' 49" N, 09° 03' 31" W), The Seven Hogs (52° 19' 33" N, 10° 01' 13" W).

National Biodiversity Network:

1 record have been used from the NBN database, but details are not given as they can be found on the NBN website: www.searchnbn.net.

**Scotland**

7 ♀♀ and juvs; on algae in water off rocks at LWM, R. S. Scott, Leicester University Expedition, 1970; Ceann Ear, Monach Islands, NW Scotland, 57⁰ 31’ N, 7⁰ 36’ W (RMNH.CRUS.1.7549).

14 ♀♀ and juvs; intertidal rocky shores among algae during low tide, P. Vieira, 2011; Carsaig, Scotland, 56° 9' N, 5° 57' W.

1 stage 8 ♂, 14 ♀♀ and juvs; intertidal rocky shores among algae during low tide, P. Vieira, 2011; Easdale, Scotland, 56° 17' N, 5° 38' W.

3 stage 6-8 ♂♂, 15 ♀♀ and juvs; intertidal rocky shores among algae during low tide, P. Vieira, 2011; Bellochantuy, Scotland, 55° 31' N, 5° 42' W.

In addition, specimens of *D. bidentata* were examined by DMH from the following locations during the period 1964-1974 (Holdich 1970, 1974): Ardrossan (55° 36' N, 04° 43' W), Clatholl (58° 10' N 00", 05° 19' 00" W).

British Natural History Museum (BMNH) collection:

Oban (56° 24' 54" N, 05° 28' 15" W), Mull (approx. 56° 26' 21" N, 06° 00' 03" W).

National Biodiversity Network:

27 records have been used from the NBN database, but details are not given as they can be found on the NBN website: www.searchnbn.net.

**Wales**

6 stage 8 ♂♂ (year 1, 5.0-6.0 mm), 1 back end stage 8 ♂, 2 stage 8 ♂♂ (year 2), 1 stage 6 ♂, 8 stage 7 ♀♀ (5.5 mm), 2 stage 8 ♀♀, 2 juvs; adults from empty *Balanus perforatus* tests on mid-shore and juveniles from mid-shore *Fucus serratus*, D.M. Holdich, 1965; St Brides Haven, Pembrokeshire, South Wales, 51⁰ 46’ 00" N, 5⁰ 6’ 00" W (RMNH.CRUS.1.7517).

3 stage 8 ♂♂ (year 2), 6 stage 8 ♂♂ (year 2), 4 stage 6 ♂, 4 stage 7 ♀♀ (5.5 mm), 3 stage 8 ♀♀, 1 juv; adults from empty *Balanus perforatus* tests on mid-shore and juveniles from mid-shore *Fucus serratus*, D.M. Holdich, 1994; St Brides Haven, Pembrokeshire, South Wales, 51⁰ 46’ 00" N, 5⁰ 6’ 00" W (RMNH.CRUS.1.7548).

In addition, specimens of *D. bidentata* were examined by DMH from the following locations during the period 1964-1975 (Holdich 1970, 1976) and subsequently: Aber-Eiddy (51° 56' 30" N, 05° 11' 55" W), Abermawr (51° 56' 17" N, 05° 12' 27" W), Broad Haven (51° 42' 05" N, 05° 09' 11" W), Caer-fai Ba (51° 52' 00" N, 05° 15' 00" W), Dinas Head (52° 01' 15" N, 04° 54' 36" W), Freshwater East (51° 38' 44" N, 04° 51' 34" W), Freshwater West (51° 38' 60" N, 05° 02' 60" W), Goodwick Harbour (51° 59' 60" N, 04° 59 '00" W), Manorbier (51° 37' 60" N, 04° 46' 60" W), Martins Heaven (51° 44' 14" N, 05° 14' 01" W), Monks Haven (51° 42' 60" N, 05° 08' 00" W), Musselwick Sands (51° 42' 60" N, 05° 12' 00" W), Nolton Haven (51° 48' 58" N, 05° 06' 27" W), Porth Colman (52° 52' 00" N, 04° 41' 00" W), Rhoscolyn (53° 15' 00" N, 04° 34' 60" W), Rhosneigre (53° 13' 00" N, 04° 30' 60" W), Sandy Haven (51° 43' 32" N, 05° 06' 41" W), Skomer (51° 43' 60" N, 05° 16' 60" W), Stackpole (51° 37' 41" N, 04° 53' 45" W), West Angle (51° 40' 60" N, 05° 04' 60" W), West Dale Bay (51° 42' 28" N, 05° 11' 19" W), Whitesand Bay (51° 53' 10" N, 05° 18' 18" W. Records for the Gower Peninsula in South Wales prior to 1961 have not been included as the isopod was eliminated from this region in the severe winter of 1961/62, and the authors have seen no new records.

National Biodiversity Network:

12 records have been used from the NBN database, but details are not given as they can be found on the NBN website: www.searchnbn.net.

**Channel Islands**

14 stage 8 ♂♂, 3 stage 7 ♀♀, 14 stage 8 ♀♀, 2 juvs; upper mid-shore crevice with tubiculous tanaids, mid and lower shore in empty *Balanus perforatus* shells and crevices, D.M.Holdich, 1982; Rocquaine Bay, Guernsey, 49⁰ 26’ N, 2⁰ 39’ W (RMNH.CRUS.1.7551).

4 stage 8 ♂♂, 3 stage ♀♀; mid shore in empty *Balanus perforatus* shells, D.M. Holdich, 1982; Petit Bot Bay, Guernsey, 49⁰ 25’ N, 2⁰ 34’ W (RMNH.CRUS.1.7552).

4 ♀♀; lower shore weed (*Chondrus crispus*), D.M. Holdich, 1982; Rocquaine Bay, Guernsey, 49⁰ 26’ N, 2⁰ 39’ W (RMNH.CRUS.1.7553).

**France**

2 stage 8 ♂♂ (6.0-6.5 mm), 1 stage 7 ♀ (5.5 mm), 1 stage 8 ♀; adults from empty *Balanus perforatus* test on mid-shore; ♀7 from mid-shore *Ascophyllum nodosum*, D.M. Holdich, 1975, shore by Roscoff Marine Station, Brittany, France, 48⁰ 43’ N, 3⁰ 59’ W (RMNH.CRUS.1.7518).

2 stage 7 ♂♂, 7 stage 8 ♂♂ (one 7 mm), 2 stage 8 ♀♀, 16 ♀♀ and juvs; algae, empty barnacle tests and crevices, mid-lower shore, D.M. Holdich, 1988; Trénez, S. Brittany, 47⁰ 47’ N, 3⁰ 42’ W (RMNH.CRUS.1.7560).

5 ♀♀ and juvs; red algae lower shore, D.M. Holdich, 1988; Trénez, S. Brittany, 47⁰ 47’ N, 3⁰ 42’ W (RMNH.CRUS.1.7561).

4 stage 7 ♂♂, 7 stage 8 ♂♂, 9 females ♀♀ and juvs; rocky mid-shore weed and crevices, D.M Holdich, 1988; Kerfanny, S. Brittany, 47⁰ 51’ N, 3⁰ 38’ W (RMNH.CRUS.1.7562).

1 stage 7 ♂, 2 stage 8 ♂♂, 2 stage 8 ♀♀, 4 ♀♀ and juvs; empty barnacle tests and mid-shore algae, D.M. Holdich, 1975; Roscoff Marine Station Brittany, 48⁰ 43’ N, 3⁰ 59’ W (RMNH.CRUS.1.7563).

2 stage 7 ♀♀; littoral, L. Deckker, 1982; Bestree Pord, Finisterre, 48⁰ 15’ N, 3⁰ 55’ W (RMNH.CRUS.1.7480).

1 juv; H. Nouvel, 1952; Isla Vete de Callo Baie de Morlaiz, not far from Roscoff, 48⁰ 43’ N, 3⁰ 53’ W (RMNH.CRUS.1.7481).

1 stage 8 ♂ (5.5 mm), 1 juv; C. Swennen, 1958; Perros-Guirec, Brittany, 48⁰ 48’ N, 3⁰ 26’ W (RMNH.CRUS.1.7482).

2 stage 8 ♂♂ (8mm and 6 mm), 1 juv; H. Nouvel, 1952; Roscoff, Brittany, 48⁰ 43’ N, 3⁰ 59’ W (RMNH.CRUS.1.7483).

1 stage 7 ♂, 1 stage 7 ♀; H. Nouvel, 1936; Brest, St Ann, 48⁰ 23’ N, 4⁰ 29’ W (RMNH.CRUS.1.7492).

Museum National d’Histoire Naturelles, Paris collection:

1 ♀; 1922; location unknown (IS 1288).

2 ♀♀; 1922; Concarneau, S. Brittany, 48⁰ 48’ N, 3⁰ 26’ W (IS 1292).

1 stage 6 ♂; 1922; Concarneau, 48⁰ 48’ N, 3⁰ 26’ W (IS 1303).

1 female; Concarneau, 48⁰ 48’ N, 3⁰ 26’ (IS 1291).

4 stage 8 ♂♂; 1922; Concarneau, 48⁰ 48’ N, 3⁰ 26’ (IS 1295).

**Spain**

4 stage 6/7 ♂♂, 1 stage 8 ♂, 6 ♀♀, 31 juvs; *Fucus vesiculosus* zone, R. Anadon, 1982; Bañugues (Asturias), 43⁰ 31’ N, 5⁰ 39’ W (RMNH.CRUS.1.7569).

15 stage 7 ♂♂, 1 stage 8 ♂, 1 stage 8 ♀, 27 ♀♀, 51 juvs; mesolittoral, P. Reboreda, 1984, 1987, 1988; Ria del Ferrol (43⁰ 29’ N, 8⁰ 13’ W), Ria de Arosa (42⁰ 34’ N, 8⁰ 53’ W), Isla Castelo (43⁰ 36’ N, 8⁰ 11’ W) (RMNH.CRUS.1.7570).

1 ♀; littoral amongst *Leathesia*, R.M.N.H., St. 0. 15, 1962; San Vincente, Peninsula del Grove, Ria del Arosa, 42⁰ 34’ N, 8⁰ 53’ W (RMNH.CRUS.17485).

1 stage 7 ♂; R.M.N.H., St. 0. 90,1967; Punta San Vincente del Grove, 42⁰ 34’ N, 8⁰ 53’ W (RMNH.CRUS.17486).

1 stage 8 ♂ (7 mm), 1 stage 7 ♀ (5 mm); littoral in *Saccorhiza*, 1962; San Vincente, 42⁰ 34’ N, 8⁰ 53’ W (RMNH.CRUS.17489).

1 stage 8 ♂, 2 stage 7 ♀♀, 1 juv; 0-1 m, 1963; San Vincente, Peninsula dell Grove, 42⁰ 34’ N, 8⁰ 53’ W (RMNH.CRUS.17491).

1 stage 8 ♂, 77 ♀♀ and juvs; intertidal rocky shores among algae during low tide, P. Vieira, 2011; Pedreira, Galicia, 43° 33' N, 8° 16' W.

1 stage 8 ♂, 121 ♀♀ and juvs; intertidal rocky shores among algae during low tide, P. Vieira, 2011; Barizo, Galicia, 43° 19' N, 8° 52' W.

6 stage 6-8 ♂♂, 32 ♀♀ and juvs; intertidal rocky shores among algae during low tide, P. Vieira, 2011; Muxía, Galicia, 43° 5' N, 9° 13' W.

No specimens available, but a clear photograph by Guerra-Garcia shows a stage 8 ♂♂ from Tarifa island, Spain, 36⁰ 00’ N, 5⁰ 36’ W. For detailed habitat information and number of individuals refer to Izquierdo et al. (2010), Guerra-García et al. (2011), Guerra-García et al. (2012), Torrecilla-Roca and Guerra-García (2012).

**Portugal**

2 stage 8 ♂♂, 45 ♀♀ and juvs; intertidal rocky shores among algae during low tide, P. Vieira, 2011; Buarcos, Portugal, 40° 10' N, 8° 54' W.

8 stage 8 ♂♂, 6 stage 8 ♀♀, 80 juvs; intertidal rocky shores among algae, in barnacles and in crevices, during low tide, P. Vieira, 2015; Buarcos, Portugal, 40° 10' N, 8° 54' W.

1 juv; intertidal rocky shores among algae during low tide, P. Vieira, 2015; Agudela, Portugal, 41° 14' N, 8° 43' W.

1 juv; intertidal rocky shores among algae during low tide, P. Vieira, 2014; Praia Vale dos Homens, Portugal, 37° 22' N, 8° 50' W.

3 juvs, intertidal rocky shores among algae during low tide, P. Vieira, 2011; Peniche, Portugal, 39° 22' N, 9° 22' W.

18 juv; among algae, F.O. Costa, 2012; Praia Norte, Portugal, 41° 41' N, 8° 50' W.

4 juv; among algae, F.O. Costa, 2014; Sines, Portugal, 38°28' N, 8°59' W.

2 juv; among algae, F.O. Costa, 2012; Vila do Conde, Portugal, 41°21' N, 8°45' W.

**Morocco (NW Africa)**

1 ♀ stage 7; Very smooth, no hairs, no keel on pleotelsonic dome, foramen right shape. Label is in Dutch. Station 30 - found 23 km from Rabat (33⁰ 58’ N, 6⁰ 50’ W), NW Morocco, Casablanca. 20 October 1974. RMNH.CRUS.1.7450.

2 stage 7 ♂♂; H. Gantès, 1949; Témara, 33⁰ 55’ N, 6⁰ 54’ W (RMNH.CRUS.1.7451).

2 stage 8 ♂♂, 1 stage 6 ♂, 19 ♀♀ and juvs; among *Laminaria*, P. Vieira, 2015; El Jadida, Morocco, 33° 14' N, 8° 28' W.

1 stage 8 ♀; intertidal rocky shores among algae during low tide, P. Vieira, 2014; Akhfenir, Morocco, 28° 6' N, 12° 3' W.

2 stage 8 ♂♂, 11 ♀♀ and juvs; intertidal rocky shores among algae during low tide, P. Vieira, 2014; Insouane, Morocco, 30° 50' N, 9° 49' W.

1 stage 8 ♂, 30 ♀♀ and juvs; intertidal rocky shores among algae during low tide, P. Vieira, 2014; Essaouire, Morocco, 31° 30' N, 9° 46' W.

***Dynamene bifida* Torelli, 1930**

**Mediterranean**

**Spain**

1 stage 7 ♂, 1 ♀; under stones 0.5 m, D.M. Holdich, 1985; Pueblo Pier, Mojacar, Spain, 37⁰ 8’ N, 1⁰ 49’ E (RMNH.CRUS.1.7574).

**France**

1 stage 8 ♂, 1 stage 7 ♂, 1 stage 8 ♀; rocky shore, between seaweed, 0-1 m, L.B. Holthuis, 1955; Banyuls Marine Laboratory, France, 42⁰ 29’ N, 6⁰ 6’ E (RMNH.CRUS.1.7526).

1 stage 8 ♂, 1 stage 7 ♂, 1 stage ♂ 6, 3 stage 7 ♀♀; 0-1m deep, near lab, L.B. Holthuis, 1955; Banyuls-sur-Mer, France 42⁰ 29’ N, 3⁰ 7’ E (RMNH.CRUS.1.7496).

5 stage 8 ♂♂ (4-6 mm), 4 stage 8 ♀♀; rocky shore, between seaweed, 0-1 m, L.B. Holthuis, 1957; Banyuls-sur-Mer, France, 42⁰ 29’ N, 6⁰ 6’ E (RMNH.CRUS.1.7498).

**Italy**

2 stage 8 ♂♂ (one back end, likely to be >7 mm total length), 2 stage 7 ♂♂, 3 ♀♀; 1.0 m under stones, D.M. Holdich, 1969; Ischia island, Bay of Naples, Italy, 40⁰ 44' N, 13⁰ 56' E (RMNH.CRUS.1.7525).

1 stage 8 ♂ (front end only), 2 stage 8 ♀♀; amongst shallow-water *Hydroides* colony, D.M. Holdich, 1967; Mergellina, Bay of Naples, Italy, 40⁰ 49’ N, 14⁰ 13’ E (RMNH.CRUS.1.7592).

**Turkey**

1 stage 8 ♂ (4.5 x 2 mm), 1 ♀ (3.2 x 1.75); intertidal and shallow-water algae and other habitats, F. Kirkim, mid-1990s; central location of Aegean coast at Izmir, (RMNH.CRUS.1.7659).

In addition, specimens of *D. bifida* were examined by DMH from Corfu (39⁰ 36’ N, 19⁰ 49’ E) during the period 1964-1970 (Holdich 1970), but no further details are available.

***Dynamene edwardsi* (Lucas, 1849)**

**Atlantic**

**Azores (Portugal)**

1 stage 8 ♂ (3 mm), 2 stage 7 ♂♂, 2 stage 8 ♀♀ (3 mm), 6 stage 7 ♀♀ (2.8 mm), 9 juvs; rocky shore with deep tide pools, Tydeman Azores Exp. 1981; CANCAP-V. Stat. 5.K15, south-east coast Corvo, south of Rosario, 39° 40’ N, 31° 07’ W (RMNH.CRUS.1.7452).

3 stage 8 ♂♂ (4 mm), 6 stage 7 ♂♂, 12 stage 7 ♀♀ (3 mm), 1 juv; Tydeman Azores Exp. 1981; CANCAP-V. Stat. 5.K10, north coast São Jorge, Faja da Caldeira, 38° 38’ N, 27° 56’ W (RMNH.CRUS.1.7453).

1 stage 8 ♂, 1 stage 7 ♂, 5 ♀♀, 1 juv, (plus one *Campecopea lusitanica*); rock flat with holes and tide pools, much algae, Tydeman Azores Exp. 1981; CANCAP-V. Stat. 5.KO2, east coast Santa Maria, Baia, S. Lourence, 36° 56’ N, 25° 06’W (RMNH.CRUS.1.7460).

1 stage 8 ♂ (3.5 mm), 1 stage 8 ♀; tide pools and fissures with strong currents, 2 m deep, Tydeman Azores Exp. 1981; CANCAP-V, Stat. 5.KO1, Formiga**s**, 37° 16’ N, 24° 47’ W (RMNH.CRUS.1.7456).

1 stage 8 ♂ (3.5 mm); rocky shore with large protected pool and several smaller pools, all connected with the sea by crevices, considerable growth of algae, Hartog and Lavaleye, 1979; Terceira, south coast, west of Angra do Heroismo, between Baia de Vila Maria and São Mateus da Calheta. Stat. 22, 38° 39’ N, 27° 15’ W (RMNH.CRUS.1.7454).

1 stage 8 ♂ (4 mm); rocky shore collecting and snorkling, Hartog and Lavaleye, 1979; Pico, south coast, Lages harbour, Azores, 38° 24’ N, 28° 15’ W (RMNH.CRUS.1.7457).

1 juv; intertidal rocky shores among algae during low tide, P. Vieira, 2014; Ponta da Ferraria, São Miguel, 37° 51' N, 25° 51' W.

2 juvs; intertidal rocky shores among algae during low tide, P. Vieira, 2014; Mosteiros, São Miguel, 37° 54' N, 25° 49' W.

**Madeira (Portugal)**

4 stage 8 ♂♂, 1 stage 7 ♀, 1 stage 8 ♀; rocky littoral/sublittoral with boulders, snorkling, ONVERSAAGD-Madeira-Morokko Exp., 1976; south coast of Madeira, Funchal west of harbour, 32° 44’ N, 16° 44’ W (RMNH.CRUS.1.7471).

1 stage 7 ♂, 1 stage 6 ♂, 2 ♀♀, 1 juv; polluted rocky littoral, tide pools, crevices, Tydeman-Madeira-Mauritania Exp., 1978; Stat 3.KO2-CANCAP-III, south coast of Madeira, Funchal**,** 32° 38’ N, 16° 56’ W (RMNH.CRUS.1.7473).

1 stage 8 ♂, 2 stage 8 ♀♀, (plus one *Cymodoce* sp.); rocky littoral, pools, shallow sublittoral, Tydeman-Madeira-Mauritania Exp. 1978; Stat. 3, KO1-CANCAP-III, south-east coast of Madeira, Caniçal, 32° 44’ N, 16° 44’ W (RMNH.CRUS.1.7474).

1 stage 8 ♂ (4 mm by 2mm); shore-collecting, snorkling and diving, depth 0-22 m, ONVERSAAGD-Madeira-Morocco Exp., 1976; Stat. 14 south-east coast of Madeira near Caniçal, 32° 44’ N, 16° 44’ W (RMNH.CRUS.1.7475).

2 stage 8 ♂♂ (4 mm by 2mm), 1 stage 7 ♂, 1 ♀; polluted rocky littoral pools, Tydeman-Madeira-Mauritania Exp., 1978; Stat 3-KO3-CANCAP III, south coast of Madeira, Funchal, west of harbour pier 32° 38’ N, 16° 58’ W (RMNH.CRUS.1.7476).

7 ♀♀ and juvs; rocky littoral, pools, shallow sublittoral, Tydemena-Madeira-Mauritania Exp., 1978; Stat. 3.KO1-CANCAP-III, south-east coast of Madeira, Caniçal, 32° 44’ N 16° 44’ W (RMNH.CRUS.1.7477).

Many specimens, including stage 8 ♂♂; rocky littoral, pools, shallow sublittoral, Tydeman-Madeira-Mauritania Exp., 1978; Stat. 3.KO1-CANCAP-III, south-east coast of Madeira, Caniça**l**, 32° 44’ N, 16° 44’ W (RMNH.CRUS.1.7478).

17 ♀♀ and juvs;, intertidal rocky shores among algae during low tide, P. Vieira, 2011; Porto dos Frades, Porto Santo, 33° 4' N, 16° 17' W.

161 ♀♀ and juvs; intertidal rocky shores among algae during low tide, P. Vieira, 2011; Reis Magos, Madeira, 32° 38' N, 16° 49' W.

2 stage 8 ♂♂, 159 ♀♀; intertidal rocky shores among algae during low tide, P. Vieira, 2011; Ponta da Cruz, Madeira, 32° 37' N, 16° 56' W.

Portuguese Museum of Natural History and Science (MUHNAC) collection:

19 ♀♀ and juvs; intertidal; EMEPC/M@rBis/Selvagens 2010 Mission Report, 2010; Selvagem Grande, 30⁰ 8’ N, 15⁰ 52’ W (M@rBis__001450; M@rBis__001452; M@rBis__001417; M@rBis__000031).

2 juvs; intertidal; EMEPC/M@rBis/Selvagens 2010 Mission Report, 2010; Selvagem Pequena, 30⁰ 2’ N, 16⁰ 1’ W (M@rBis__000267; M@rBis__000929).

**Canary Islands (Spain)**

2 ♀♀; empty barnacle tests amongst tufted coralline algae on mid-shore lava rocks, D. M. Holdich, 2002; Playa Blanca, Lanzarote, 28⁰ 51’ N, 13⁰ 49’ W (RMNH.CRUS.1.7559).

1 stage 8 ♂; rocks, muddy, polluted littoral, Tydeman-Selvagens-Canary Is. Exp. 1980; Sta. 4.KO2: CANCAP IV, Las Palmas - north coast of Gran Canaria, 28° 9’ N, 15° 26’ W (RMNH.CRUS.1.7463).

1 stage 8 ♂ (4 mm); rocky littoral, Tydeman-Cancap-II Canary Is. Exp. 1977; Stat. K13, west coast of Fuerteventura near Punta Jandia, 28° 4’ N, 14° 30’ W (RMNH.CRUS.1.7464).

1 ♀; rocks, tide pools, sandy bay, sea-grass, depth to 5 m, Tydeman - Selvagens-Canary Is. Exp. 1980; Stat. KO6: CANCAP IV, Arinaga, east coast of Gran Canaria, 27° 51’ N, 15° 24’ W (RMNH.CRUS.1.7466).

2 stage 8 ♂♂ (4.5 mm by 2 mm), one ♀; rocky shore, tide pools, shallow sandy bay, 0-2 m, Tydeman-Selvagens-Canary Is. Exp. 1980; Stat 4. K12: CANCAP IV, Arrecif - south-east coast of Lanzarote, 28° 57’ N, 13° 33’ W (RMNH.CRUS.1.7467).

1 stage 7 ♂; rocky shore with tide flat and pools, depth 0-4 m, Tydeman-Selvagens-Canary Is. Exp. 1980; Stat. 4.K13 CANCAP IV, Rada de Arrieta - east coast of Lanzarote, 29° 9’ N, 13° 25’ W (RMNH.CRUS.1.7468).

3 stage 8 ♂♂, 1 stage 8 ♀; littoral sheltered rocky coast, rockpools and skindiving to 6 m, CANCAP-II: Tydeman Canary Is. Exp. 1977; Stat K2, Puerto de Mogan, Gran Canaria, 27° 49’ N, 15° 50’ W (RMNH.CRUS.1.74693).

93 ♀♀ and juvs; intertidal rocky shores among algae during low tide, P. Vieira, 2011; La Fajana, La Palma, 28° 50' N, 17° 47' W.

31 ♀♀ and juvs; intertidal rocky shores among algae during low tide, P. Vieira, 2011; La Salemera, La Palma, 28° 34' N, 17° 45' W.

1 stage 8 ♂, 78 ♀♀ and juvs; intertidal rocky shores among algae during low tide, P. Vieira, 2011; El Faro, La Palma, 28° 27' N, 17° 51' W.

25 stage 6-8 ♂♂, 282 ♀♀ and juvs; intertidal rocky shores among algae during low tide, P. Vieira, 2011; Bañaderos, Gran Canaria, 28° 8' N, 15° 32' W.

2 stage 8 ♂♂, 99 ♀♀ and juvs; intertidal rocky shores among algae during low tide, P. Vieira, 2011; Caleta, Gran Canaria, 28° 9' N, 15° 41' W.

2 stage 6-8 ♂♂, 63 ♀♀ and juvs; intertidal rocky shores among algae during low tide, P. Vieira, 2011; Playa Melenara, Gran Canaria, 27° 59' N, 15° 22' W.

2 juvs; intertidal rocky shores among algae during low tide, P. Vieira, 2015; Arenas Blancas, El Hierro, 27°46' N, 18° 7' W.

4 juvs; intertidal rocky shores among algae during low tide, P. Vieira, 2015; Los Sargos, El Hierro, 27°47' N, 18° 0' W.

3 juvs; intertidal rocky shores among algae during low tide, P. Vieira, 2015; Los Cristianos, Tenerife, 28° 2' N, 16°42' W.

1 stage 7 ♂, 5 juvs; intertidal rocky shores among algae during low tide, P. Vieira, 2015; Mal Paso, Tenerife, 28°24' N, 16°17' W.

**Portugal**

14 stage 8 ♂♂, t33 stage 8 ♀♀, 10 ♀♀; upper shore sandstone crevices (with *Campecopea hirsuta*) and mid-shore barnacles, D. M. Holdich, 1981; Lagos, Algarve, Portugal, 37⁰ 6’ N, 8⁰ 40’ W (RMNH.CRUS.1.7567).

1 stage 8 ♂, 1 ♀, 1 juv; amongst mussels on breakwater at mid-tide, D.M. Holdich, 1981; Villamoura, Algarve, Portugal, 37⁰ 5’ N, 8⁰ 7’ W (RMNH.CRUS.1.7568).

11 ♀♀ and juvs; intertidal rocky shores among algae during low tide, P. Vieira, 2011; Peniche, Portugal, 39° 22' N, 9° 22' W.

11 ♀♀ and juvs; intertidal rocky shores among algae during low tide, P. Vieira, 2011; Lagos, Algarve, Portugal, 37° 5' N, 8° 40' W.

1 stage 8 ♂, 1 juv; intertidal rocky shores among algae during low tide, P. Vieira, 2011; Arrifes, Algarve, Portugal, 37° 4' N, 8° 16' W.

6 stage 6-8 ♂♂, 25 ♀♀ and juvs; intertidal rocky shores among algae during low tide, P. Vieira, 2011; Ingrina, Algarve, Portugal, 37° 2' N, 8° 52' W.

2 juvs; intertidal rocky shores among algae during low tide, P. Vieira, 2014; Praia Vale dos Homens, Portugal, 37° 22' N, 8° 50' W.

1 juv; intertidal rocky shores among algae during low tide, P. Vieira, 2014; Buarcos, Portugal, 40° 10' N, 8° 54' W.

1 juv; intertidal rocky shores among algae during low tide, P. Vieira, 2014; Berlengas, Portugal, 39° 24' N, 9° 30' W.

1 juv; among algae, F.O. Costa, 2014; Sines, Portugal, 38° 28' N, 8° 59' W.

**Spain**

1 ♀; intertidal rocky shores among algae during low tide, P. Vieira, 2011; Muxía, Galicia, Spain, 43° 5' N, 9° 13' W.

No specimens available, but a clear photograph by Guerra-Garcia shows a stage 8 ♂♂ from Tarifa island, Spain, 36⁰ 00’ N, 5⁰ 36’ W. For detailed habitat information and number of individuals refer to Izquierdo et al. (2010), Guerra-García et al. (2011), Guerra-García et al. (2012), Torrecilla-Roca et al. (2012).

**Morocco (NW Africa)**

1 stage 8 ♂; from fouling organisms on side of ship, H. Zibrowius, 1980s; Tanger (Tangiers) Harbour Tangiers, 35⁰ 53’ N, 5⁰ 30’ W (RMNH.CRUS.1.7571).

1 stage 8 ♂, 29 juvs and ♀♀ and juvs; among algae in pools, P. Vieira, 2015; Arzila, Morocco, 35° 27' N, 6° 2' W.

1 stage 8 ♂, 1 juv; intertidal rocky shores among algae, P. Vieira, 2015; El Jadida, Morocco, 33° 14' N, 8° 28' W.

3 stage 6-8 ♂♂, 16 ♀♀ and juvs; intertidal rocky shores among algae during low tide, P. Vieira, 2014; Tarfaya, Morocco, 27° 54' , 12° 57' W.

2 juvs; intertidal rocky shores among algae during low tide, P. Vieira, 2014; Insouane, Morocco, 30° 50' N, 9° 49' W.

**Mauritania (Western Africa)**

Monod’s (1923) description of *D. hanseni* from Port Étienne (now Nouadhibou) (20⁰ 56’ N, 17⁰ 2’ W) is clearly *D. edwardsi*. Monod’s specimen (Museum National d’Histoire Naturelles, Paris collection) was seen and verified by DMH.

**Mediterranean**

**Spain**

1 stage 8 ♂, 1 ♀; no details of habitat, H. Zibrowius, 1980s; Alicante, Spain, 38⁰ 20’ N, 0⁰ 29’ E (RMNH.CRUS.1.7572).

1 stage 7 ♀ (4.5 mm); no habitat details, L.B. Holthuis, 1949; Baai van Cadaqués, Casa Zariguiey, Spain, 42⁰ 17’ N, 3⁰ 15’ E (RMNH.CRUS.1.7494).

1 stage 7 ♀; no habitat details, J. Castello, 1983; Cala Morell, Menorca, Spain, 40⁰ 3’ N, 3⁰ 53’ E (RMNH.CRUS.1.7577).

1 stage 7 ♂; no habitat details, J. Castello, 1984; Cala Olivera, Ibiza, Spain, 38⁰ 57’ N, 1⁰ 24’ E (RMNH.CRUS.1.7581).

**France**

9 stage 8 ♂♂, 2 stage 7 ♂♂, 1 stage 6 ♂, 15 ♀♀, 10 juvs; 5-6 m, H. Zibrowius, 1980s; Ponteau, nr Marseille, 43⁰ 22’ N, 5⁰ 76’ E, and Marseille, France - 43⁰ 17’ N, 5⁰ 22’ E (RMNH.CRUS.1.7586).

1 stage 7 ♂, 1 stage 8 ♀, 4 ♀♀, 2 juvs; from shallow-water *Cystoseira*, I. Gordon, 1952; Banyuls-sur-Mer, France, 42⁰ 29’ N, 3⁰ 7’ E (RMNH.CRUS.1.7587).

4 stage 8 ♂♂, 2 stage 7 ♂♂, 3 ♀♀, 1 stage 8 ♀ (one male 5.5 mm by 3 mm, one female 4 mm by 2.2 mm); 0-1 m depth, 1957; Port Vendres, south of Perpignan, France, 43⁰ 31’ N, 3⁰ 7’ E (RMNH.CRUS.1.7497).

2 stage 8 ♂♂, intertidal on artificial hard surfaces in docks, A. Marchini, 2014; La Grande Motte, France, 43°33' N 4° 5' W.

**Monaco**

1 stage 7 ♂, one juv.; 1-2 m, 1952; Baiede Garnoles, Monaco, 43⁰ 44’ N, 7⁰ 23’ E (RMNH.CRUS.1.7500).

**Italy**

2 stage 8 ♂♂ (5.0 mm), 2 stage 7 ♂♂, 3 ♀♀; shallow water crevices and empty barnacle tests, D.M. Holdich, 1967; Mergellina Harbour, Naples, Italy, 40⁰ 49' N, 14⁰ 13' E (RMNH.CRUS.1.7522).

1 stage 7 ♂, 2 ♀♀, 5 juvs; no habitat details, H. Zibrowius, 1980s; Vado Ligure, Gulf of Genova, Italy, 44⁰ 16’ N, 8⁰ 26’ E (RMNH.CRUS.1.7590).

2 stage 8 ♂♂, 5 stage 7 ♂♂, 4 stage 8 ♀♀, 22 ♀♀ and juvs; from shallow-water *Hydroides* colony, *Cystoseira* and *Sargassum*, D. M. Holdich, 1967; V. Galloti, Mergellina, Bay of Naples, Italy, 40⁰ 49’ N, 14⁰ 13’. Two ♀♀ added to this vial; no habitat details, E. Fresi, 1967; Capri island, Bay of Naples, Italy, 40⁰ 33’ N, 14⁰ 13’ E (RMNH.CRUS.1.7593).

12 stage 8 ♂♂, 8 ♀♀; intertidal on artifical hard surfaces in docks, J. Ferrario and A. Marchini, 2014; Genoa Harbour, Italy, 44⁰ 24’ N, 8⁰ 55’ E (RMNH.CRUS.1.7595).

10 stage 8 ♂♂, 5 stage 8 ♀♀, 2 ♀♀; intertidal on artifical hard surfaces in docks, J. Ferrario and A. Marchini, 2014; Santa Margherita Ligure docks, Italy, 44⁰ 19’ N, 9⁰ 12’ E (RMNH.CRUS.1.7596).

2 stage 8 ♂♂, 2 stage 7 ♂♂, 2 ♀♀; walls of canal, R. Sconfietti, 1982-83; Basino di San Marco and Porto di Lido, Venice, Italy, 45⁰ 26’ N, 12⁰ 18’ E (RMNH.CRUS.1.7524).

11 stage 8 ♂♂, 4 stage 7 ♂♂, 3 stage 8 ♀♀; 5 females, 1 juv; walls of canal, R. Sconfietti, 1982-83; Basino di San Marco and Porto di Lido, Venice, Italy, 45⁰ 26’ N, 12⁰ 18’ E (RMNH.CRUS.1.7597).

4 stage 8 ♂♂, 2 stage 7 ♂♂, 1 stage 6 ♂, 5 stage 8 ♀♀, 6 ♀♀, 1 juv; 0.5 m, brown and red algae on rocks, U. Schieke and E. Fresi, 1968, 1969, 1970; Carta Romana, Castello and below Ecological Laboratory, Ischia island, Bay of Naples, Italy, 40⁰ 44’ N, 13⁰ 57’ E (RMNH.CRUS.1.7603).

5 stage 8 ♂♂, 2 stage 6 ♂♂, 8 juvs; intertidal on artificial hard surfaces in docks, J. Ferrario and A. Marchini, 2014; Marina of Porto Retondo, Sardinia, Italy, 41° 1' N 9°32' W.

1 stage 8 ♂; intertidal on artificial hard surfaces in docks, J. Ferrario and A. Marchini, 2014; Marina of Castelsardo, Sardinia, Italy, 40° 54' N 8°42' W.

1 stage 8 ♂, 1 stage 6 ♂, 1 stage 8 ♀, 28 juvs; intertidal on artificial hard surfaces in docks, J. Ferrario and A. Marchini, 2013; Harbour of Leghorn, Tuscany, Italy, 43°33' N 10°17' W.

5 stage 8 ♂♂, 3 stage 6 ♂♂, 2 stage 8 ♀♀, 11 juvs; intertidal on artificial hard surfaces in docks, P. J. Ferrario and A. Marchini, 2013; Harbour of la Spezia, Liguria, Italy, 44° 6' N 9°54' W.

**Croatia**

2 stage 8 ♂♂, intertidal on artifical hard surfaces in docks, P. M. Maric, 2014; Marina Kornati, Croatia, 43°56' N 15°26' W.

**Greece**

1 stage 8 ♂; no habitat details; H. Zibrowius, 1980s; Rhodes island, Greece, 36⁰ 25’ N, 28⁰ 13’ E (RMNH.CRUS.1.7616).

2 stage 8 ♂♂, 3 stage 7 ♂♂, seven ♀♀; under rocks in 20-30 cm water, D.M. Holdich, 2001; Lindos, Rhodes island, Greece, 36⁰ 5’ N, 28⁰ 5’ E **(**RMNH.CRUS.1.7642).

3 stage 7 ♂♂, 10 ♀♀ and juvs; variety of algae, e.g. *Cystoseira*, *Sargassum*, from 0.5 m – 33 m, D.A. Jones, 1967; Emborios Bay and Cathedral Rock, Chios island, Greece, 38⁰ 11’ N, 26⁰ 1’ E (RMNH.CRUS.1.7646).

1 stage 7 ♂, 3 ♀♀; shallow water algae, D.A. Jones, 1967; Emborios Bay, Chios island, Greece, 38⁰ 11’ N, 26⁰ 1’ E (RMNH.CRUS.1.7532).

**Turkey**

2 stage 8 ♂♂ (4.5 x 2.3 mm, 5 x 2.5 mm), 1 stage 8 ♀; 1 stage 7 ♀ (4.5 x 2.2 mm); intertidal and shallow-water algae and other habitats, F. Kirkim, 1995; Aegean coast at Izmir, 38⁰ 28' N, 27⁰ 6' E (RMNH.CRUS.1.7658).

**Malta**

2 stage 8 ♂♂, one ♀; 0.5-1 m, rocky seabed, L. Bonnici, 2010; Birzebbugh, Malta, 35⁰ 47’ N, 14⁰ 31’ E (RMNH.CRUS.1.7663).

British Natural History Museum (BMNH) collection:

1 stage 7 ♂; fenders; Valetta Harbour, 35⁰ 54’ N, 14⁰ 30’ E.

**Israel**

2 stage 8 ♂♂, 1 stage 7 ♂, 2 ♀♀; littoral algae from rocky shore, L. Fischelson, 1976; Acre north of Haifa, Israel, 32⁰ 55' N, 35⁰ 4' E (RMNH.CRUS.1.7523).

1 stage 7 ♂, 4 ♀♀, 1 juv; littoral algae, L. Fischelson, 1976; Rosh HaNikra, Haifa, Israel, 33⁰ 5' N, 35⁰ 6' E (RMNH.CRUS.1.7647).

1 stage 8 ♂, 3 stage 6/7 ♂♂, 3 ♀♀ (one 3.5 x 1.5 mm), 5 juvs; algae on rocky shore (*Ulva*, *Jania*), T. Haran, 1977-78; Mikhmoret, Israel, 32⁰ 24' N, 34⁰ 52' E (RMNH.CRUS.1.7648).

21 stage 8 ♂♂ (4 x 1.75 mm – 3 x 1.5 mm), 17 stage 6/7 ♂♂, 1 stage 8 ♀39 ♀♀, 4 juvs; littoral algae (*Colpomenia*, *Cystoseira*, *Hypnea*, *Laurencia*, *Jania*), from rocky shore, L. Fischelson, 1976; Acre, north of Haifa, Israel, 32⁰ 55' N, 35⁰ 4' E (RMNH.CRUS.1.7651).

1 stage 8 ♂; littoral algae (*Acanthophora*), L. Fischelson, 1976; Bat-Yam, south of Tel Aviv, Israel, 32⁰ 5 N, 34⁰ 48' E (RMNH.CRUS.1.7653).

**Egypt**

2 stage 7 ♂♂, 5 ♀♀; no habitat details, M.M. Atta, 1981; Alexandria, Egypt, 31⁰ 9’ N, 29⁰ 55’ E (RMNH.CRUS.1.7666).

**Tunisia**

No specimen available, but a clear 2009 photograph by R. García, shows a stage 8 ♂ from Tunis, Tunisia, 36⁰ 50’ N, 10⁰ 14’ E.

**Algeria**

Naesea edwardsi Lucas, 1849.

No specimens available, but description by Lucas (1849) is clearly of this species. Precise co-ordinates are not known, so approximate ones from Algiers harbour were used: 36⁰ 48’ N, 3⁰ 13’ E.

**Suez Canal**

Glynn’s (1972) record for the Suez Canal at Tis’ A, which is close to Suez and the entrance to the Gulf of Suez, is clearly of this species based his figures. 29⁰ 58’ N, 32⁰ 32’ E.

**Red Sea**

**Egypt-Israel**

1 stage 8 ♂ (4 x 1.75 mm), 1 ♀, one juv.; littoral algae (*Padina*, *Galaxauma*), L. Fischelson, 1976; Eilat Port, Israel, 29⁰ 31' N, 34⁰ 56' E, and Dahab (Egypt) further south on north coast of Gulf of Aqaba, 28⁰ 30' N, 34⁰ 30' E (RMNH.CRUS.1.7665).

***Dynamene magnitorata* Holdich, 1968**

**Atlantic**

**Azores (Portugal)**

1 stage 8 ♂ (damaged), 1 stage 7 ♂, 4 ♀♀, 3 juvs; A. Costa (University of the Azores, Ponta Delgada, S. Miguel), 1995 and M. Jones M. (2 juveniles) (University of Plymouth), 1996; São Miguel Island, 37⁰ 46’ N, 25⁰ 29’ W (RMNH.CRUS.1.7555).

3 stage 8 ♀♀; from shallow sub-tidal empty *Megabalanus azoricus* tests (along with *Eurydice affinis* and amphipods), scuba diving, A. Costa, 2010; São Miguel Island, 37⁰ 46’ N, 25⁰ 29’ W (RMNH.CRUS.1.7556).

2 ♀♀; rocky cove with tide pools, cobble beach, subtidal algae, Tydeman. Azores Exp. 1981, CANCAP-V. Stat. 5.KO3, 1981; south coast of Santa Maria, 36° 57’ N, 25° 07’ W (RMNH.CRUS.1.7455).

1 stage 8 ♂ (4 mm), 1 stage 7 ♀ (3 mm), 1 juv; rocky shore collecting, snorkling, Hartog and Lavaleye, 1979. Pico, south coast, Lages harbour, 38° 24’ N, 28° 15’ W (RMNH.CRUS.1.7457).

2 stage 7 ♂♂, 1 stage 6 ♂, 3 ♀♀, 7 juvs, plus 1 *Cymodoce* sp; Depth approx. 10-20 m, sheltered bay, Tydeman Azores Exp, CANCAP-V. Stat. 5. DO1, 1981; south coast of São Miguel, 37° 43’ N, 25° 30’ W (RMNH.CRUS.1.7461).

2 ♀♀, 4 juvs; Tydeman Azores Exp. 1981, CANCAP-V. Stat. 5. DO7, 1981; rocky coast, south of harbour, south-east coast Faial near Horta, 38° 31’ N, 28° 37’ W (RMNH.CRUS.1.7462).

1 stage 8 ♂; Tydeman Azores Exp. 1981, CANCAP-V. Stat. 5.D11, 1981; west entrance to small bay, north coast of Flores, 39° 31’ N 31° 12’ (RMNH.CRUS.1.7459).

1 stage 8 ♂ (4 mm), 1 stage 7 ♀; depth 20 m, cobbles with algae, van Veen grab; Tydeman Azores Exp. 1981, CANCAP-V. Stat. 5.116, 1981; north of Sao Jorge, 38° 38’ N 27° 55’ (RMNH.CRUS.1.7458).

1 stage 8 ♂, 42 ♀♀ and juvs; intertidal rocky shores among algae during low tide, P. Vieira, 2014; Porto Martins, Terceira, 38° 40' N, 27° 3' W.

2 juvs; intertidal rocky shores among algae during low tide, P. Vieira, 2015; Praia Formosa, Santa Maria, 36° 56' N, 25° 5' W.

2 juvs; intertidal rocky shores among algae during low tide, P. Vieira, 2014; Cinco Ribeiras, Terceira, 38° 40' N, 27° 19' W.

1 stage 6 ♂, 22 ♀♀ and juvs; intertidal rocky shores among algae during low tide, P. Vieira, 2015; Ribeira Chã, São Miguel, 37° 42' N, 25° 29' W.

British Natural History Museum collection:

♂♂, ♀♀, juvs, lot of samples; habitat and site data apparently available, 1959; Terceira Is, 38° 43’ N, 27° 13’ W – general co-ordinates for island.

Museum National d’Histoire Naturelle, Paris collection

3 stage 8 ♂♂, 1 stage 8♀, 2 ♀♀; J. Charcot Biacores, 1971; Azores – no other details, IS 770.

1 stage 8 ♂, 2 ♀♀; J. Charcot Biacores, 1971; Azores – no other details, IS 780.

**Madeira (Portugal)**

1 ♀; Tydeman - Selvagens-Canary Is. Exp. Stat. 4. K26: CANCAP IV, Porto Santo (this is an island north of Madeira), SW coast Baixo, 33° 04’ N, 16° 20’ W (RMNH.CRUS.1.7465).

Numerous ♀♀ and juvs, plus some *Cymodoce* sp. and other crustaceans; rocky littoral pools, shallow sublittoral, Tydeman - Madeira-Maritania Exp. Stat. 3.KO1-CANCAP-III, SE coast of Madeira, Caniçal, 1978; 32° 44’ N, 16° 44’ W (RMNH.CRUS.1.7472).

1 juv; intertidal rocky shores among algae during low tide, P. Vieira, 2011; Porto dos Frades, Porto Santo, 33° 4' N, 16° 17' W.

**Canary Islands (Spain)**

2 stage 8 ♂♂ (4 mm x 1.75 mm); surface dip net, CANCAP-II: Tydeman Canary Is. Exp. Stat. 17, 1977; south of Fuerteventura, Punta Jandia, 27° 39’ N, 14° 22’ W (RMNH.CRUS.1.7468).

1 juv; intertidal rocky shores among algae during low tide, P. Vieira, 2011; La Fajana, La Palma, 28° 50' N, 17° 47' W.

2 stage 8 ♂♂, 1 juv; intertidal rocky shores among algae during low tide, P. Vieira, 2011; El Faro, La Palma, 28° 27' N, 17° 51' W.

**England**

1 ♀; red seaweed in rockpool, M. Storey, 2011; Newton’s Cove, Weymouth, Dorset 50⁰ 40’ N, 2⁰ 30’ W (RMNH.CRUS.1.7547).

**Channel Islands**

4 stage 6/7 ♂♂, 2 stage 8 ♂♂ (one 5 x 2.2 mm), 5 ♀♀; lower shore weed (*Chondrus crispus*) sponge, *Halichondria*, channels (males), D. M. Holdich, 1982; Guernsey, L’Eree, Rocquaine Bay, 49⁰ 26’ N, 2⁰ 39’ W (RMNH.CRUS.1.7553).

2 stage 8 ♂♂ (one 4 mm long), 8 stage 7 ♂♂, 18 ♀♀, 5 juvs; rubbings from red algae on lower shore, D. M. Holdich, 1982 Petit Bot Bay, Guernsey, 49⁰ 25’ N, 2⁰ 34’ W (RMNH.CRUS.1.7554).

**France**

3 stage 8 ♂♂ (5.0 mm), 1 stage 8 ♀, 2 stage 7 ♀♀; rocky shore crevices, D. M. Holdich, 1975; Roscoff Marine Station, 48⁰ 43’ N, 3⁰ 59’ W (RMNH.CRUS.1.7520).

1 stage 8 ♂, 3 stage 7 ♂♂, 3 ♀♀; red algae on lower shore, D. M. Holdich, 1988; Trénez, S. Brittany, 47⁰ 47’ N, 3⁰ 42’ W (RMNH.CRUS.1.7561).

1 ♀; rocky mid-shore weed and crevices, D. M. Holdich, 1988; Kerfanny, S. Brittany, 47⁰ 47’ N, 3⁰ 43’ W (approx.) (RMNH.CRUS.1.7562).

12 stage 8 ♂♂, 3 stage 7 ♂♂, 4 stage 8 ♀♀, 8 ♀♀ and juvs; empty barnacle test and mid-shore algae, D. M. Holdich, 1975; Roscoff Marine Station shore, Brittany, 48⁰ 43’ N, 3⁰ 59’ W (RMNH.CRUS.1.7564).

1 stage 8 ♂, stage 7 ♂♂, many juveniles, H. Nouvel, 1939; Bisdarz, Roscoff, 48⁰ 43’ N, 3⁰ 59’ (approx.) (RMNH.CRUS.1.7484).

1 stage 8 ♂, 1 stage 7 ♀; H. Nouvel, 1952. Grève Santec, Perarhidy, 48⁰ 41’ N, 3⁰ 58’ W (RMNH.CRUS.1.7487).

1 stage 7 ♀; H. Nouvel, 1952; Roscoff, Térèris, NW France, 48⁰ 43’ N, 3⁰ 59’ W (approx.) (RMNH.CRUS.1.7488).

Museum National d’Histoire Naturelles, Paris collection

1 ♀; Th Monod; Guitec, NW France, IS 678.

In addition, specimens of *D. magnitorata* were examined by DMH from the following locations during the period 1964-1975 (Holdich 1970, 1976) and subsequently:

Argenton (48° 31' 41" N, 04° 46' 41" W), Barfleur (49° 40' 04" N, 01° 15' 24" W), Brignognan (48° 40' 20" N, 04° 18' 49" W), Isles de Glénans (47° 17' 37" N, 03° 12' 28" W), Trégaster (48° 50' 32" N, 03° 31' 06" W).

**Portugal**

2 stage 8 ♂♂, 10 stage 7 ♂♂, 7 ♀ and juvs; sub-tidal algae, D. M. Holdich, 1981; Amação de Pêra, Algarve, 37⁰ 6’ N, 8⁰ 21’ W (RMNH.CRUS.1.7565).

1 stage 7 ♂, 6 ♀♀ and juvs; sub-tidal algae, D. M. Holdich, 1981; Lagos, Algarve, 37⁰ 6’ N, 8⁰ 40’ W (RMNH.CRUS.1.7566).

2 stage 8 ♂♂, 9 stage 6 ♂♂, 192 ♀♀ and juvs; intertidal rocky shores among algae during low tide, P. Vieira, 2011; Peniche, Portugal, 39° 22' N, 9° 22' W.

5 ♀♀ and juvs; intertidal rocky shores among algae and in crevices during low tide, P. Vieira, 2014; Peniche, Portugal, 39° 22' N, 9° 22' W.

1 juv; intertidal rocky shores among algae during low tide, P. Vieira, 2011; São Pedro Moel, Portugal, 39° 45' N, 9° 1' W.

1 stage 8 ♂, 39 ♀♀ and juvs; intertidal rockyshores among algae during low tide, P. Vieira, 2011; Lagos, Algarve, Portugal, 37° 5' N, 8° 40' W.

52 ♀♀ and juvs; intertidal rocky shores among algae during low tide, P. Vieira, 2011; Arrifes, Algarve, Portugal, 37° 4' N, 8° 16' W.

1 juv; among algae, F.O. Costa, 2013; Arrabida, Portugal, 38° 28' N, 9° 59' W.

10 juv; among algae, F.O. Costa, 2012; Praia Norte, Portugal, 41° 41' N, 8° 50' W.

51 juvs; intertidal rockyshores among algae during low tide, P. Vieira, 2011; Buarcos, Portugal, 40° 10' N, 8° 54' W.

2 juvs; intertidal rockyshores among algae during low tide, P. Vieira, 2014; Berlengas, Portugal, 39° 24' N, 9° 30' W.

**Spain**

1 ♀; mesolittoral, Reboreda, P. (University of Santiago de Compostela, Spain), 1984, 1987, 1988; Puerto Sou, Ria de Noi, 42⁰ 43’ N, 8⁰ 59’ W (RMNH.CRUS.1.7570).

1 stage 8 ♂, 1 stage 7 ♂ 7, 1 stage 7 ♀ (4 mm); 1963; San Vincent, 43⁰ 22’ N, 4⁰ 23’ W (RMNH.CRUS.1.7479).

1 stage 7 ♂, plus 1 stage 6 ♂ and 1 ♀ *D. bidentata*; littoral, 1962; Jidorio Pectregoso, west of Is. de Arosa’ Exc. R.M.N.H. Sta. 0. 75, 42⁰ 33’ N, 8⁰ 51’ W (RMNH.CRUS.17490).

3 stage 8 ♂♂, 607 ♀♀ and juvs; intertidal rocky shores among algae during low tide, P. Vieira, 2011; Pedreira, Galicia, Spain, 43°33' N, 8°16' W.

1 stage 8 ♂, 48 ♀♀ and juvs; intertidal rockyshores among algae during low tide, P. Vieira, 2011; Barizo, Galicia, Spain, 43° 19' N, 8° 52' W.

4 stage 6-8 ♂♂, 132 ♀♀ and juvs; intertidal rocky shores among algae during low tide, P. Vieira, 2011; Muxía, Galicia, Spain, 43° 5' N, 9° 13' W.

No specimens available, but a clear photograph by Guerra-Garcia shows a stage 8 ♂♂ from Tarifa island, Spain, 36⁰ 00’ N, 5⁰ 36’ W. For detailed habitat information and number of individuals refer to Izquierdo et al. (2010), Guerra-García et al. (2011), Guerra-García et al. (2012), Torrecilla-Roca et al. (2012).

**Morocco (NW Africa)**

4 ♀♀ and juvs; among algae in pools, P. Vieira, 2015; Arzila, Morocco, 35° 27' N, 6° 2' W

Museum National d’Histoire Naturelle, Paris collection:

2 stage 8 ♂♂, 5 ♀♀; T. Monod; Fedhala, nr Casablanca, 33⁰ 41’ N, 7⁰ 22’ W, IS 680.

**Mediterranean**

**Spain**

3 stage 8 ♂♂; 0-1 m, H. Zibrowius, 1986; Alicante, 38⁰ 20’ N, 0⁰ 29’ E (RMNH.CRUS.1.7573).

2 ♂ stage 8, 1 ♀ stage 8, 1 ♀ stage 7; 0.0 m – 20.0 m on a variety of algae, C. Catellanos et al. (University of Alcalá, Spain), 1991-1993; Chafarinas Islands, off Mediterranean Morocco, Spanish Territory, 35⁰ 11' N, 2⁰ 25' E (RMNH.CRUS.1.7521)

**Monaco**

1 stage 7 ♂ (damaged); 5-6 m, H. Nouvel, 1971; en face de port Fonteveille, 43⁰ 43’ N, 7⁰ 25’ E (RMNH.CRUS.1.7499).

**Italy**

1 stage 8 ♂, 1 ♀ (back end); F. Maggiore, 1975; Ischia Porto 40⁰ 44’ N, 13⁰ 57’ E (RMNH.CRUS.1.7610). Labelled as *D. bidentata* (see Maggiore and Fresi 1984).

**Egypt**

3 stage 8 ♂♂, 2 stage 7 ♂♂, 2 ♀♀, 2 juvs; M. M. Atta, 1981; Alexandria, 31⁰ 9’ N, 29⁰ 55’ E (RMNH.CRUS.1.7668).

**Tunisia**

Museum National d’Histoire Naturelle, Paris collection:

3 stage 8 ♂♂; T. Monod; Tunis? IS 678.

***Dynamene tubicauda* Holdich, 1968**

**Mediterranean**

**Italy**

2 stage 8 ♂♂, 1 stage 8 ♀, 2 ♀♀, 2 juvs; 10 metres, E. Fresi & U. Schieke, 1968; Ischia island, Naples, Italy, 40⁰ 44' N, 13⁰ 56' E (RMNH.CRUS.1.7531).

2 stage 8 ♂♂; 12 m and 20 metres on *Halimeda*, U. Schieke & E. Fresi, E., 1967; Banco S. Croce, Sorrento, Bay of Naples, Italy, 40⁰ 37’ N, 14⁰ 22’ E (RMNH.CRUS.1.7594).

3 stage 8 ♂♂, 2 stage 8 ♀♀, 8 ♀♀, 4 juvs; on *Dictyota*, *Vidalia* and *Halimeda* at 10.5 metres (very muddy) (D.S. Pancrazio); 30 metres on sand and coralline fragments (Secca di ischia), U. Schieke & E. Fresi, 1967, 1968, 1969, 1970; Ischia island, Italy, 40⁰ 44’ N, 13⁰ 57’ E (RMNH.CRUS.1.7611).

3 stage 8 ♂♂, 3 stage 7 ♂♂, 1 ♀, one juvenile; 2-5 metres, H. Zibrowius, 1980; Elba island, Italy, 42 ⁰ 47’ N, 10⁰ 08’ E (RMNH.CRUS.1.7598).

From rock scappings and algae at 5-10 metres around Sicily (Italy), Lombardo (1984) recorded 1 stage 8 ♂ from Isola Lachea (37⁰ 33’ N, 15⁰ 9’ E) (Catania), 3 stage 8 ♂♂ from Brucoli (37⁰ 16’ N, 15⁰ 11’ E) (Siracusa), and 1 ♀ from Calaberdardo (36⁰ 52’ N, 15⁰ 85’ E) (Siracusa). The present authors have not seen the specimens for the present study but the published drawings are clearly of this species.

**Malta**

4 stage 8 ♂♂; *Posidonia oceanica* meadow at 12 m, J. A. Borg, 1998; Mellieha Bay, Malta, 35⁰ 58’ N, 14⁰ 21’ E (RMNH.CRUS.1.7664).

***Dynamene* sp.**

**Aegean**

1 stage 8 ♂; found in stomach of black scorpionfish *Scorpaena porcus*, M. Băcescu, 1982; N.W. Aegean, 40⁰ N, 25⁰ E (approx.) (RMNH.CRUS.1.7533).

1 stage 8 ♂; found in stomach of black scorpionfish *Scorpaena porcus*, M. Băcescu, 1982; N.W. Aegean, 40⁰ N, 25⁰ E (approx.) (RMNH.CRUS.1.7660).
